# Spinal Cord Injury: A Systematic Review and Network Meta-Analysis of Therapeutic Strategies Based on 15 Types of Stem Cells in Animal Models

**DOI:** 10.3389/fphar.2022.819861

**Published:** 2022-03-14

**Authors:** Zhizhong Shang, Ruirui Wang, Dongliang Li, Jinlei Chen, Baolin Zhang, Mingchuan Wang, Xin Wang, Pingping Wanyan

**Affiliations:** ^1^ The First Clinical Medical School of Lanzhou University, Lanzhou, China; ^2^ Chengren Institute of Traditional Chinese Medicine, Lanzhou, China; ^3^ Department of Spine, Changzheng Hospital, Naval Medical University, Shanghai, China; ^4^ Gansu University of Chinese Medicine, Lanzhou, China; ^5^ The Second Hospital of Lanzhou University, Lanzhou, China

**Keywords:** stem cell, spinal cord injury, therapeutic strategies, animal studies, systematic review, network meta-analysis

## Abstract

**Objective:** The optimal therapeutic strategies of stem cells for spinal cord injury (SCI) are fully explored in animal studies to promote the translation of preclinical findings to clinical practice, also to provide guidance for future animal experiments and clinical studies.

**Methods:** PubMed, Web of Science, Embase, CNKI, Wangfang, VIP, and CBM were searched from inception to September 2021. Screening of search results, data extraction, and references quality evaluation were undertaken independently by two reviewers.

**Results and Discussion:** A total of 188 studies were included for data analysis. Results of traditional meta-analysis showed that all 15 diverse types of stem cells could significantly improve locomotor function of animals with SCI, and results of further network meta-analysis showed that adipose-derived mesenchymal stem cells had the greatest therapeutic potential for SCI. Moreover, a higher dose (≥1 × 106) of stem cell transplantation had better therapeutic effect, transplantation in the subacute phase (3–14 days, excluding 3 days) was the optimal timing, and intralesional transplantation was the optimal route. However, the evidence of current animal studies is of limited quality, and more high-quality research is needed to further explore the optimal therapeutic strategies of stem cells, while the design and implementation of experiments, as well as measurement and reporting of results for animal studies, need to be further improved and standardized to reduce the risk when the results of animal studies are translated to the clinic.

**Systematic Review Registration**: [website], identifier [registration number].

## Introduction

Spinal cord injury (SCI) can result in many serious consequences, such as paralysis, sensorimotor dysfunction, urinary incontinence, and gastrointestinal dysfunction, and the age of over half of the patients is 16–30 years old (Kooijmans et al., 2017). Moreover, cervical SCI accounts for approximately 55% of all SCI cases, and its mortality rate is as high as 10% in the first year, with a life expectancy of only 10–15 years after injury (Vawda et al., 2012). Globally, according to the World Health Organization (WHO), about 250,000–500,000 people suffer from SCI annually, with approximately 11,000–20,000 new cases each year in the United States alone (Organization and Society, 2013; Cox, 2018).

The loss of massive neurons and glial cells, demyelination, cavities, and glial scar formation result from the death of nerve cells within 12 h to a few weeks after SCI (Guest et al., 2005; Barnabé-Heider and Frisén, 2008). The current clinical therapies such as spinal decompression surgery, treatment for spasticity, and rehabilitation therapy can merely alleviate symptoms, however, it is difficult to promote the regeneration of damaged nerves and the recovery of motor function (Ashammakhi et al., 2019). Altogether SCI has become a major health issue of global concern.

Recently, the development of molecular and regenerative medicine as well as insights into the pathophysiology of SCI has brought new hope for the treatment of SCI (Mosley et al., 2017). Among these, cell therapy has become a focus in the field of SCI because of its unique potential for neuroprotection, angiogenesis, immunomodulation, and tissue regeneration (Ashammakhi et al., 2019; Guo et al., 2019). In particular, various stem cells, such as neural stem cells (NSCs), mesenchymal stem cells, and others, can differentiate into neural cells and glial cells, and thus exhibit great therapeutic potential in preclinical studies by replacing damaged neurons, promoting remyelination of axons, promoting angiogenesis, bridging cysts or cavities, reducing inflammatory factors, and so on, and promoting functional recovery of SCI (De Feo et al., 2012; Mothe and Tator, 2012; Ashammakhi et al., 2019). However, their further applications are limited by the disadvantages of low survival rate, easy migration away from the site of injury, hyperproliferation, formation of ectopic stem cells or tumors, differentiation into cells that do not require or cannot be regenerated, and abnormal axon formation (Ashammakhi et al., 2019).

At present, stem cell therapy for SCI remains controversial. For example, multiple studies have shown NSCs are the best choice to promote functional recovery of patients with SCI (Abematsu et al., 2010; Amemori et al., 2013). However, Nutt et al. (2013) viewed that NSCs have limited capacity to repair SCI. In addition, bone marrow mesenchymal stem cells (BMSCs) have shown promising potential in the treatment of SCI because of their low immunogenicity and high availability, while they can secrete a number of molecules including nerve growth factor (NGF), brain-derived neurotrophic factor (BDNF), and vascular endothelial growth factor (VEGF) (Huang et al., 2021). However, some studies have shown that BMSCs do not show better effects in improving motor function in patients with SCI than conventional therapy (Karamouzian et al., 2012). In contrast, umbilical cord-derived mesenchymal stem cells (UCMSCs) are more effective in promoting motor recovery than BMSCs (Kaner et al., 2010).

Although many previous animal studies (Yousefifard et al., 2016; Assinck et al., 2017; Nagoshi and Okano, 2017) and meta-analysis results showed that (Oliveri et al., 2014; Muthu et al., 2020) stem cell transplantation improved the motor function in animals with SCI, there were still some limitations: (1) In previously published systematic reviews, stem cells of different types, sources, transplant doses, transplant routes, and transplant timings were unified as experimental groups to compare with the placebo groups, subgroup analysis was not performed. Although it is concluded that stem cells have potential advantages, this “comprehensive” analysis method makes the results more heterogeneic when combined, thus affecting the authenticity of the meta-analysis results, which makes it difficult to provide reference for clinical trials (Oliveri et al., 2014). 2) In the published studies, only the data of the endpoint of follow-up were selected for final analysis, which was difficult to test the therapeutic effect of stem cells in the whole process, and the endpoint of follow-up of different studies was quite different, so the rationality of the combination of results at different time points was questionable (Oliveri et al., 2014; Yousefifard et al., 2016). 3) It has been suggested that the transplantation timing of stem cells was the key factor that determines their targeting effect (Chhabra and Sarda, 2017a), and the optimal transplantation dose and type of stem cells were major issues that promoted further development of stem cell therapy for SCI (Abbaszadeh et al., 2018). Although nearly a thousand animal experiments of stem cell therapy for SCI have been published, including various stem cells and their transplantation doses, timings, and routes, few studies have explored which kind of dose, route, and timing of stem cells are the best for the treatment of SCI (Zholudeva and Lane, 2019). In addition, preliminary clinical trials of stem cells for SCI have been conducted. However, its unsatisfactory therapeutic effect and serious complications limit further large-scale clinical trials (Hu et al., 2021). The main reason why patients do not achieve the expected results is that the optimal repair strategy of stem cells is still unclear. At the same time, it is even more unrealistic to explore the optimal repair strategy clinically due to safety and ethical issues.

Therefore, we intend to comprehensively collect the animal studies of stem cell therapy for SCI at home and abroad, explore the real effects of different stem cell therapies through traditional meta-analysis and network meta-analysis in node-wise manner, and at the same time further explore the optimal stem cell type for repairing SCI and its dose, timing, and route of transplantation. Our results will be of immense value in reducing the risk of translation of animal experimental findings to the clinic, avoiding waste of experimental resources, and facilitating the development of animal experiments and clinical research in the future.

## Materials and Methods

### Inclusion and Exclusion Criteria

#### Subjects

The results of previous experiments based on animal models of SCI (cats, dogs, and monkeys) while the conducted clinical trials were disappointing. The pathophysiology of glial scars and cysts produced during SCI in rodents (mainly rats) is more similar to humans, while at the same time is less costly and more standardized, making them the most commonly used SCI animal models to date (Tator, 1995; Kjell and Olson, 2016). Therefore, we included rat SCI models without restricting the animal strain and modeling modality.

#### Interventions

Stem cells, without restriction to their species and source.

#### Control

(1) Positive control: comparison of different routes, doses, and timings of transplantation between the same stem cells, or contrasts between different stem cells. (2) Negative control: normal saline, PBS, vehicle, cultural medium, blank, DMSO, DMEM.

#### Outcome

Basso–Beattie–Bresnahan (BBB) locomotor rating scale (Basso et al., 1996). BBB score was from 0 points (no visible hind limb movement change) to 21 points (continuous plantar gait and continuous coordination with forelimb movement change; toe continuous grip when moving forward; when the foot is in contact with the ground or raised, the main posture is parallel to the body; the tail is continuously raised and the trunk is continuously stable), which could directly reflect the recovery of motor function in rats. The higher the score, the better the recovery of motor function of the rats.

Most studies reported the BBB score of rats with SCI within 8 weeks of follow-up. However, there was no research on staging the recovery stage of SCI in rats in the previous research or systematic review published at present. To avoid duplication of data and maximize the utilization of the obtained data, we performed a rational selection of data based on the recovery of motor function and inflammatory response in rats after SCI.

The data were selected as follows. The early phase of cellular inflammation consists mainly of neutrophils (peaking at 1 d after injury), macrophages/microglia (peaking at 7 d after injury), and T cells (peaking at 9 d after injury) (Beck et al., 2010). In addition, studies on rats showed that transplanting macrophages with an M2 phenotype promoted nerve regeneration and improved functional recovery after SCI in rats, also, macrophages peaked again at 60 days post-injury (Beck et al., 2010; Nakajima et al., 2012; Miron et al., 2013). Among the included studies, most were transplanted stem cells immediately after injury. Therefore, we selected data from studies at 1 and 8 weeks (the period with the most robust inflammatory response) after stem cell therapy for analysis. In addition, the glial scar composed of astrocytes can form a physical barrier and express molecules that inhibit axonal growth. Three weeks after injury is a critical period for the “scar” maturation of astrocytes (Kjell and Olson, 2016). Therefore, we selected study data from week 3 after stem cell therapy for analysis. Previous studies have shown that the recovery of motor function of rats after SCI showed a plateau at about 5 weeks (Osaka et al., 2010; Morita et al., 2016). Thus, we selected study data from week 5 after stem cell therapy for analysis.

In summary, we selectively included data from weeks 1, 3, 5, and 8 after stem cell therapy for the final analysis. Our data were chosen based on the lack of staging criteria for the recovery of motor function in rats after stem cell transplantation in the most rational way, which may also provide some references for future studies. However, the scientific and rationality of staging methods need to be further verified by establishing a standardized SCI model.

#### Type of Study

Control studies were included, there were no restrictions on the blind method.

### Search Strategy

Electronic search of PubMed, Ovid-Embase, Web of Science, China National Knowledge Infrastructure (CNKI), Chinese Scientific Journal Database (CSJD-VIP), Wanfang Database, and China Biomedical Literature Database (CBM) databases was performed from inception to September 2021. Additionally, we searched reference lists of identified articles and published meta-analysis and reviews. The search terms are shown: (Spinal cord injury OR Spinal injury OR Spinal Cord Trauma OR Spinal Cord Transection OR Spinal Cord Laceration OR Post-Traumatic Myelopathy OR Spinal Cord Contusion) AND (Stem cell OR Stem cells). See Supplementary Table S1 for full search strategies of each database.

### Literature Screening and Data Extraction

Two trained researchers selected the papers and extracted the data in strict accordance with the inclusion/exclusion criteria and cross-checked them. In case of disagreement, a third party would decide. Data were extracted according to the pre-established full-text data extraction checklist, including: (1) Basic information: author, year, type of study; sex, age, body weight, sample size, and modeling methods of the animals; type, source, dose, route, timing of transplantation of stem cell; and intervention measures in the control group. (2) Outcomes: BBB score.

### Risk of Bias Assessment

Based on SYRCLE’s risk of bias tool for animal studies (Hooijmans et al., 2014), two trained researchers independently evaluated and cross-checked the inherent risk of bias in the included studies, covering selection bias, implementation bias, measurement bias, follow-up bias, reporting bias, and other bias from a list of 10 questions or tools. A difference in opinions was negotiated or decided by a third party. The answer to the assessment questions (tools) should be either “yes” which indicated low risk of bias, or “no” which indicated high risk of bias. For unclear items, an answer with “unclear” was assigned.

### Statistical Analysis

STATA 16.0 Software was used for the traditional meta-analysis of the data. Weighted mean difference (WMD) was used as the effect analysis statistic for continuous variables, and 95% confidence intervals (CI) were provided for each effect. Heterogeneity of results between studies was assessed by a χ2 test, and the significant level for heterogeneity test was *p* = 0.1. Meanwhile, combine with I^2^ to quantitatively judge the size of heterogeneity. If there was no statistical heterogeneity among the results, the fixed effect model was adopted for meta-analysis. Otherwise, the sources of heterogeneity were further analyzed. After excluding the influence of obvious clinical heterogeneity, the random effects model was used for meta-analysis. Significance level for tests was set at 0.05.

The statistics of Bayesian meta-analysis were performed using GeMTC-0.14.3 software. GeMTC-0.14.3 software used Markov chain-Monte Carlo (MCMC) method prior to an evaluation of the data based on the Bayesian model, to realize network meta-analysis. The initial iteration was set to 50,000 times. Model fit was assessed and compared between fixed- and random-effects models using the deviance information criterion (DIC). A consistency model was used for network meta-analysis, and *p* < 0.05 was considered statistically significant. The inconsistency between direct and indirect evidence was assessed by using the node-splitting model, which could calculate the difference between direct and indirect evidence. At the same time, the potential scale reduced factor (PSRF) was used to evaluate the convergence of the results. When the PSRF value is close to or equal to 1, the convergence is complete, the model has good stability, and the conclusion of analysis is reliable. We performed data preprocessing by network group command of STATA 16.0 software to draw a web plot for comparison among various interventions and detect publication bias.

Through the comprehensive analysis of the included studies, we only conducted a network meta-analysis of NSCs and mesenchymal stem cells from bone marrow, fat, and umbilical cord, which are widely studied and have great therapeutic potential (Assinck et al., 2017). The small number of stem cell studies of other types are susceptible to individual studies to produce small sample bias, while there is large selection bias, reporting bias, and measurement bias, it is rare to make reliable conclusions. Therefore, we only conducted traditional meta-analysis on them.

We comprehensively compared the most effective stem cells for SCI by network meta-analysis, and then, performed network meta-analysis of this stem cell in the transplantation timings after SCI [acute phase, ≤ 3 days; subacute phase, ≤ 14 days; chronic phase, > 14 days (Rowland et al., 2008; Yousefifard et al., 2019)], the transplantation doses (high dose, ≥1 × 10^6^; low dose, <1 × 10^6^) (Yousefifard et al., 2016), and the transplantation routes (spinal cord, tail vein) to obtain the optimal therapeutic strategy of this stem cell.

## Results

### Literature Search Results

**FIGURE 1:**
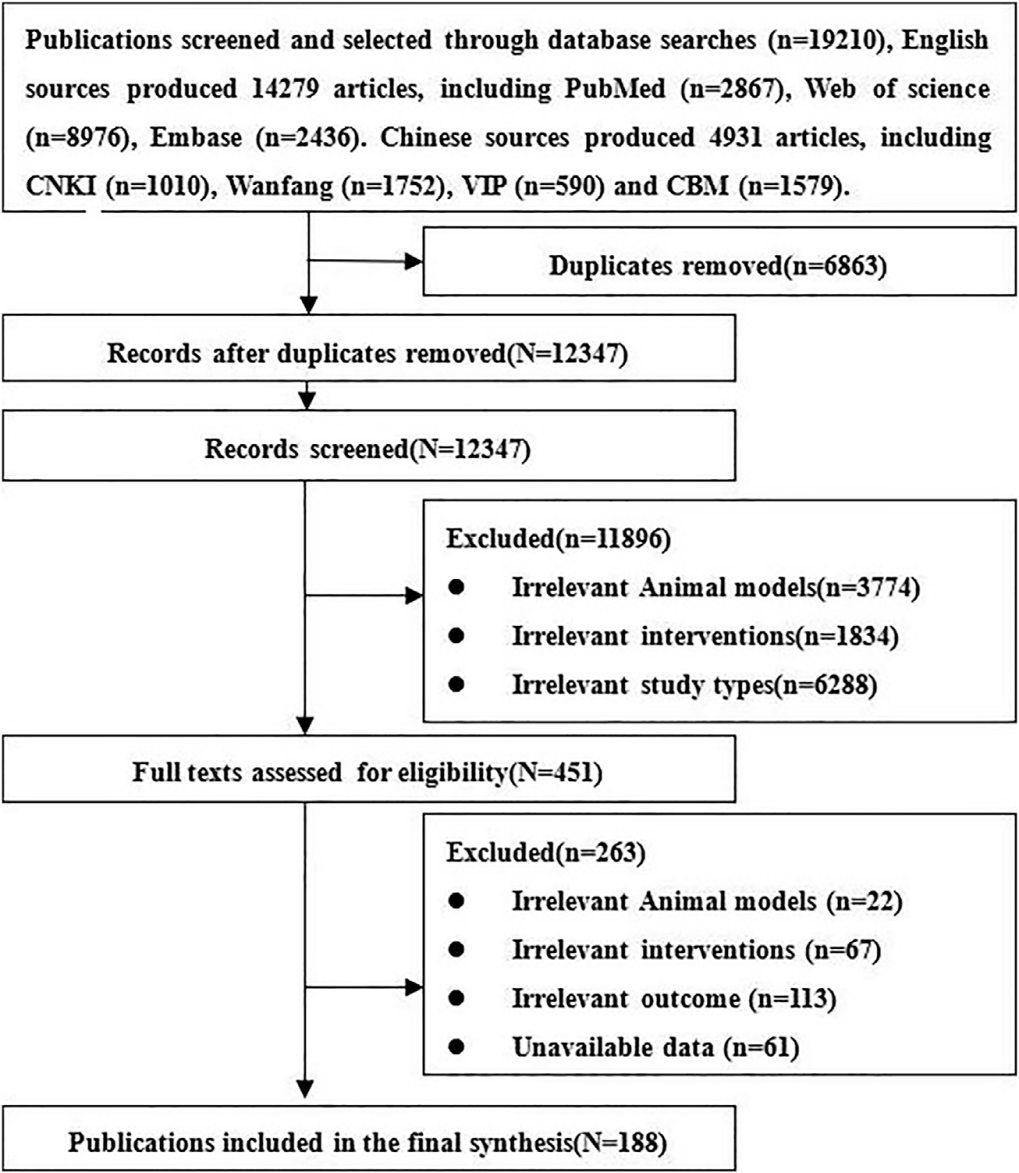
Flowchart of references—screening process.

We obtained 19,210 relative references, including 4931 Chinese references and 14,279 English references. After excluding duplicates and those not meeting inclusion criteria, 188 references of stem cell therapy for SCI were finally included, including 153 English and 35 Chinese references. The references screening process is listed in Figure 1.

### Basic Information of the Included Studies

**FIGURE 2:**
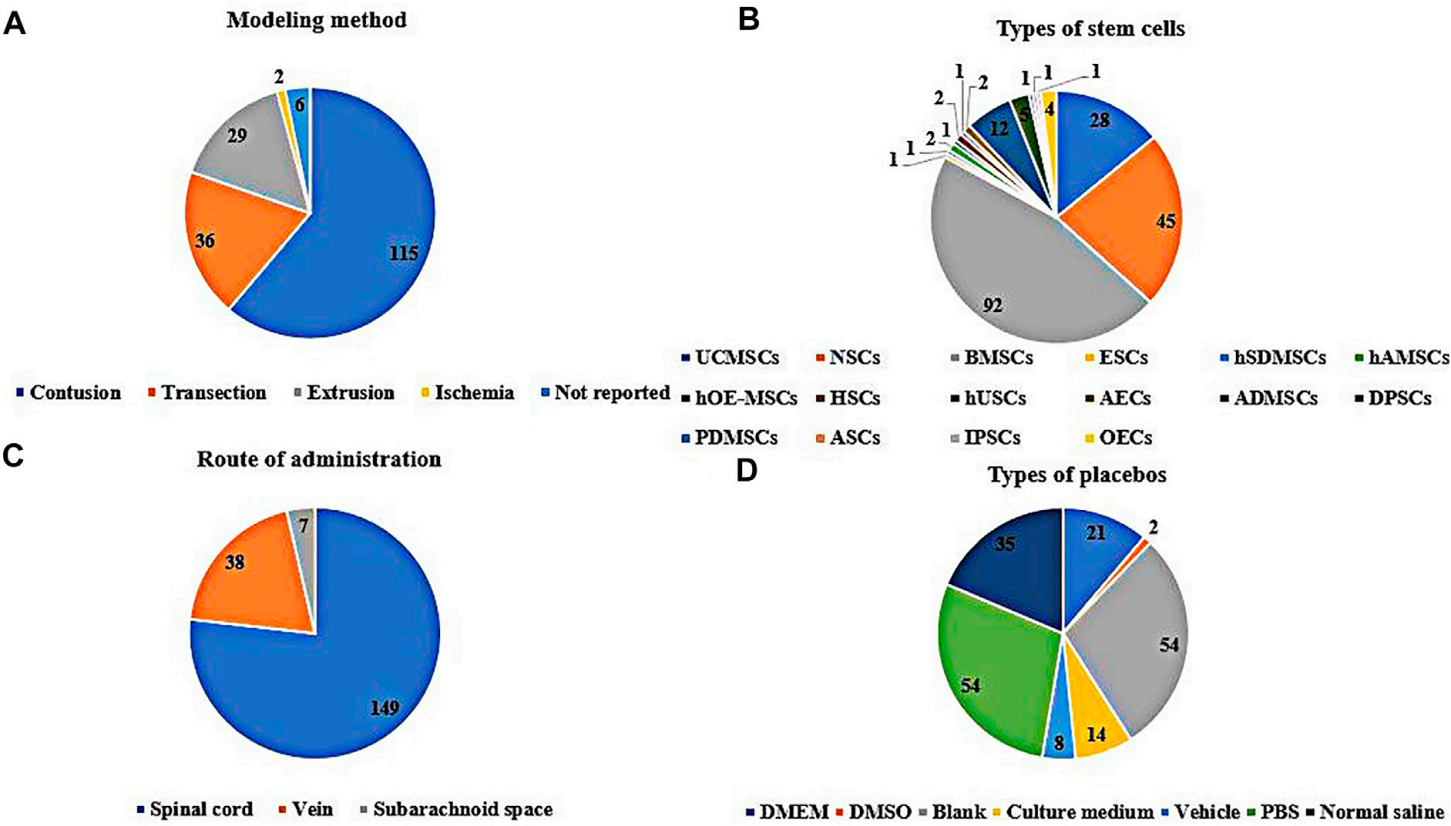
Basic information for inclusion in the study [**(A)**: The modeling method rats. **(B)**: Types of stem cells. **(C)**: Route of administration of stem cells. **(D)**: Specific measures in the placebo group].

In the included studies, 166 were randomized controlled trials, and 22 were controlled studies. The breeds of rats included albino rats (1 study), CBH-rnu/Arc (Athymic Nude) rats (1 study), Fischer 344 rats (2 studies), Long-Evans hooded rats (2 studies), SD rats (127 studies), Wistar rats (53 studies), and the breed of rats was not reported in two studies. The sex of the rats included male (70 studies), female (80 studies), half male and half female (13 studies), and the sex of the rats was not reported in 25 of these studies. The weight of the rats ranged from 70 to 400 g, age ranged from 3 to 16 weeks, sample size ranged from 6 to 141, the mode of modeling included extrusion (29 studies), contusion (115 studies), ischemia (2 studies), transection (36 studies), and 6 studies did not report specific modeling modalities, see Figure 2A. The included studies involved 15 kinds of stem cells, including bone marrow mesenchymal stem cells (BMSCs, 92 studies), neural stem cells (NSCs, 45 studies), umbilical cord mesenchymal stem cells (UCMSCs, 28 studies), adipose-derived mesenchymal stem cells (ADMSCs, 12 studies), amniotic epithelial cells (AECs, 2 studies), adult stem cells (ASCs, 1 study), dental pulp stem cells (DPSCs, 5 studies), embryonic stem cell (ESCs, 1 study), human amniotic mesenchymal stem cells (hAMSCs, 2 studies), human olfactory ecto-mesenchymal stem cells (hOE-MSCs, 1 study), hematopoietic stem cells (HSCs, 1 study), human skin-derived mesenchymal stromal cells (hSDMSCs, 1 study), human urine-derived stem cells (hUSCs, 1 study), placental-derived mesenchymal stem cells (PDMSCs, 1 study), induced pluripotent stem cells (IPSCs, 1 study), see Figure 2B. Sources of stem cells include autologous, allogeneic, xenogeneic stem cells, routes of transplantation include intralesional (149 studies), vein (38 studies), subarachnoid space (7 studies), see Figure 2C. Doses of transplantation ranged from 1 × 10^4^ to 1 × 10^8^. Negative controls included normal saline, PBS, vehicle, cultural medium, blank, DMSO, DMEM, see Figure 2D. The basic information of the included studies is shown in Supplementary Table S2.

### Results of Risk-of-Bias Assessment

**FIGURE 3:**
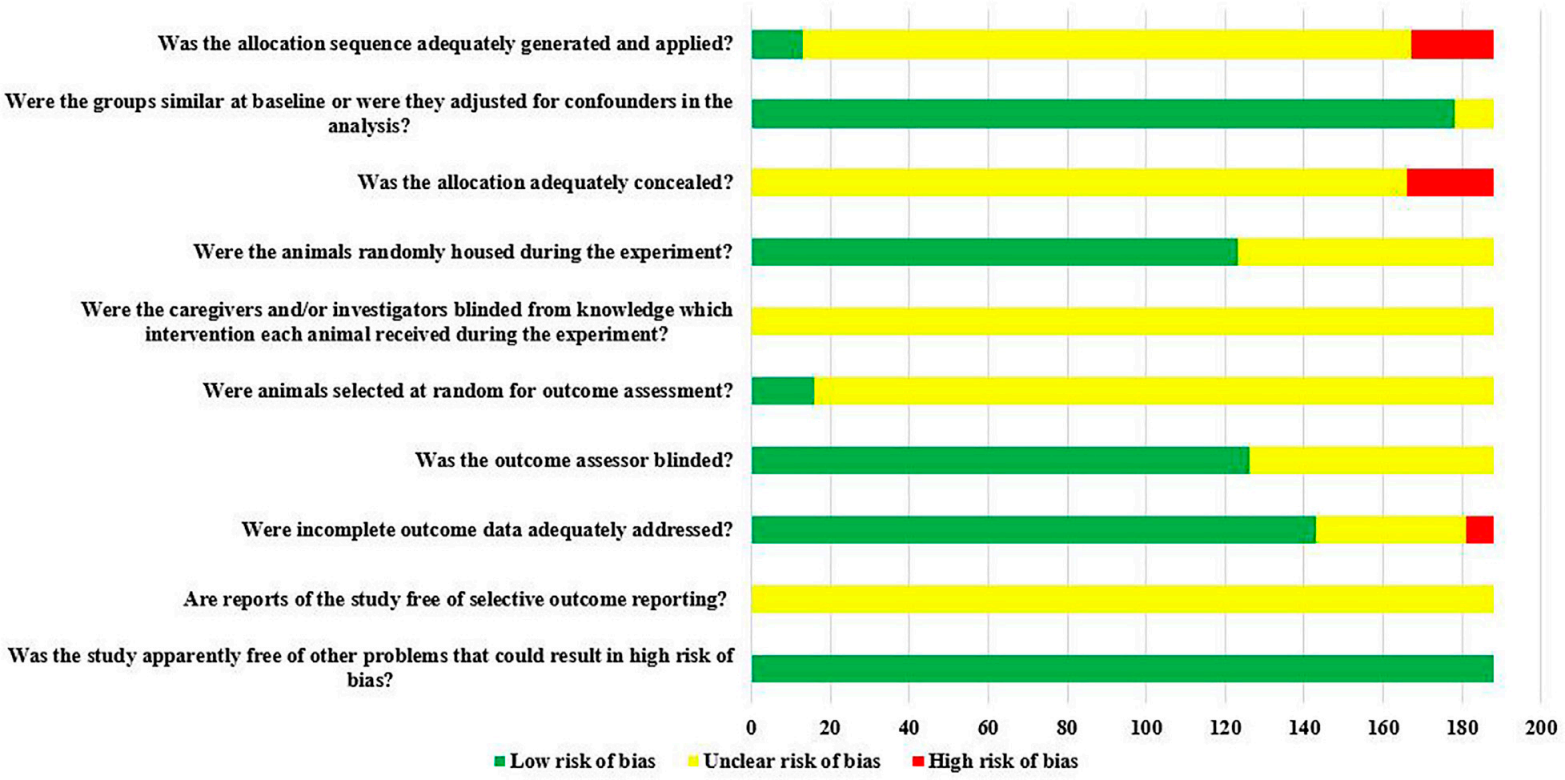
The results of the risk of bias assessment.

Of the 188 studies included, there were 166 RCTs, however, only 7.83% (13/166) reported randomization of laboratory animals using a random number table or computerized randomization, but it did not report whether concealed grouping was implemented. There was 97.68% (178/188) of the studies that clearly reported the balance of baseline characteristics such as age, sex, and body weight of rats; 65.43% (123/188) of studies reported randomized placement of rats during the experimental period. Due to the limited information provided by the included studies, it was not possible to judge whether they were blind to animal breeders and/or investigators. Only 8.51% (16/188) of studies reported randomly selecting animals at the time of outcome measurement. Blinding of outcome assessors was applied in 67.02% (126/188) of studies. The rats in 76.06% (143/188) of the studies were all included in the final analysis. All studies did not have access to the protocol, but all expected results were clearly reported. The risk of bias assessment for all studies is detailed in Figure 3.

### Results of a Traditional Meta-Analysis of 15 Different Types of Stem Cells

#### First Week After Stem Cell Therapy

Of the 188 included studies, 157 studies reported the recovery of motor function in rats at week 1 after stem cell therapy, including 13 types of stem cells (except hOE-MSCs and hUSCs). The results of meta-analysis based on the random effects model showed that the BBB scores of all 13 types of stem cells groups were significantly higher than those of the negative control group, and the difference was statistically significant, see Supplementary Table S3.

#### Third Week After Stem Cell Therapy

Of the 188 included studies, 146 studies reported the recovery of motor function in rats at week 3 after stem cell therapy, including all 15 types of stem cells. The results of meta-analysis based on the random effects model showed that the BBB scores of all stem cell groups were significantly higher than those of the negative control group, and the difference was statistically significant, see Supplementary Table S4.

#### Fifth Week After Stem Cell Therapy

Of the 188 included studies, 94 studies reported the recovery of motor function in rats at week 5 after stem cell therapy, including 12 types of stem cells (except PDMSCs, AECs, and hUSCs). The results of meta-analysis based on the random effects model showed that the BBB scores of all 12 types of stem cells groups were significantly higher than those of the negative control group, and the difference was statistically significant, see Supplementary Table S5.

#### Eighth Week After Stem Cell Therapy

Of the 188 included studies, 56 studies reported the recovery of motor function in rats at week 8 after stem cell therapy, including 10 types of stem cells (except PDMSCs, AECs, hUSCs, ESCs, and HSCs). The results of meta-analysis based on the random effects model showed that the BBB scores of all 10 types of stem cell groups were significantly higher than those of the negative control group, and the difference was statistically significant, see Supplementary Table S6.

### Results of Network Meta-Analysis of Four Types of Stem Cells

#### First Week After Stem Cell Therapy

**FIGURE 4:**
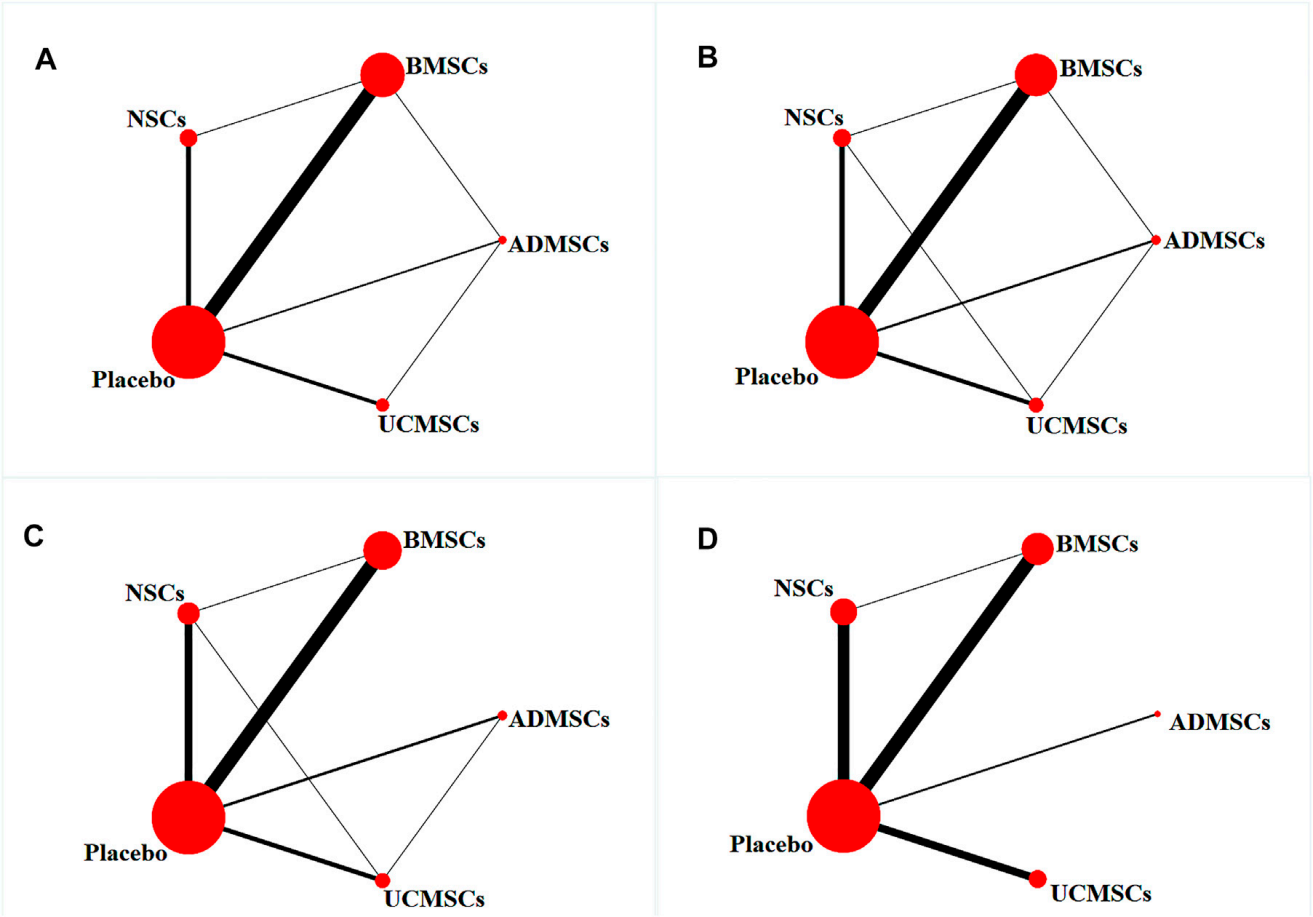
Evidence network diagram [Circle size represents sample size involved; thickness of the line segment represents the number of studies involving both interventions. **(A)** The first week; **(B)** the third week; **(C)** the fifth week; **(D)** the eighth week].

**FIGURE 5:**
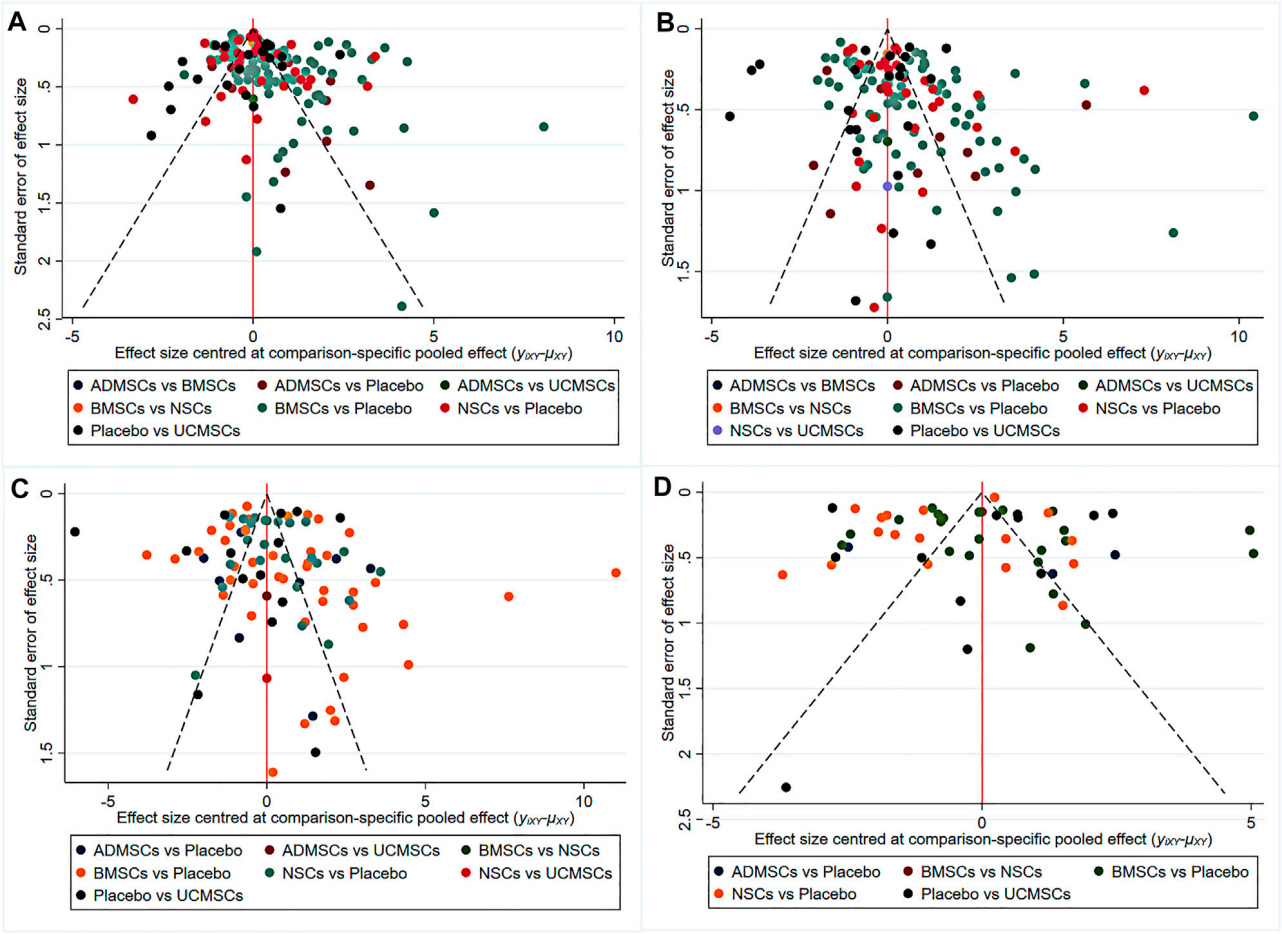
The comparison-correction funnel plot [**(A)** The first week. **(B)** The third week. **(C)** The fifth week. **(D)** The eighth week].

A total of 146 studies were included for network meta-analysis. The evidence network showed that there was no direct comparison between BMSCs vs. UCMSCs, NSCs vs. UCMSCs, and NSCs vs. ADMSCs, and there were few studies on direct comparison between other types of stem cells. At the same time, the number of studies on BMSCs was the largest, see Figure 4A. Consistent with the results of traditional meta-analysis, the results of network meta-analysis indicated that rats had significantly higher BBB scores in stem cell groups compared to negative controls. However, the differences in BBB scores of rats between the four types of stem cells were not statistically significant, see Table 1. The comparison-correction funnel plot was basically symmetrical, suggesting that there was less possibility of publication bias and small sample effect, see Figure 5A. Rank ordering results showed that UCMSCs might be the most effective stem cells for SCI, see Supplementary Table S7.

**TABLE 1:** Network meta-analysis results 1 week after stem cell therapy.

ADMSCs				
0.03 (−1.28, 1.35)	BMSCs			
0.62 (−0.83, 2.04)	0.58 (−0.24, 1.42)	NSCs		
1.47 (0.21, 2.73)	1.44 (1.03, 1.86)	0.85 (0.13, 1.57)	Placebo	
−0.19 (−1.69, 1.30)	−0.22 (−1.23, 0.78)	−0.81 (−1.96, 0.34)	−1.66 (−2.58, −0.74)	UCMSCs

#### Third Week After Stem Cell Therapy

A total of 133 studies were included for network meta-analysis. The evidence network showed that there was no direct comparison between BMSCs vs. UCMSCs, NSCs vs. ADMSCs, and there were few studies on direct comparison between other types of stem cells. At the same time, the number of studies on BMSCs was the largest, see Figure 4B. Consistent with the results of traditional meta-analysis, the results of network meta-analysis indicated that rats had significantly higher BBB scores in stem cell groups compared to negative controls. However, the differences in BBB scores of rats between the four types of stem cells were not statistically significant, see Table 2. The comparison-correction funnel plot was asymmetric, suggesting that there may be publication bias and small sample effects, see Figure 5B. Rank ordering results showed that ADMSCs might be the most effective stem cells for SCI, see Supplementary Table S8.

**TABLE 2:** Network meta-analysis results 3 weeks after stem cell therapy.

ADMSCs				
0.02 (−1.63, 1.69)	BMSCs			
0.41 (−1.41, 2.27)	0.39 (−0.69, 1.51)	NSCs		
3.02 (1.46, 4.61)	3.00 (2.42, 3.58)	2.61 (1.67, 3.54)	Placebo	
0.11 (−1.76, 1.95)	0.07 (−1.15, 1.27)	−0.32 (−1.76, 1.09)	−2.93 (−3.99, −1.89)	UCMSCs

#### Fifth Week After Stem Cell Therapy

A total of 86 studies were included for network meta-analysis. The evidence network showed that there was no direct comparison between BMSCs vs. UCMSCs, BMSCs vs. ADMSCs, and NSCs vs. ADMSCs, and there were few studies on direct comparison between other types of stem cells. At the same time, the number of studies on BMSCs was the largest, see Figure 4C. Consistent with the results of traditional meta-analysis, the results of network meta-analysis indicated that rats had significantly higher BBB scores in stem cell groups compared to negative controls. However, the differences in BBB scores of rats between the four types of stem cells were not statistically significant, see Table 3. The comparison-correction funnel plot was asymmetric, suggesting that there may be publication bias and small sample effects, see Figure 5C. Rank ordering results showed that ADMSCs might be the most effective stem cells for SCI, see Supplementary Table S9.

**TABLE 3:** Network meta-analysis results 5 weeks after stem cell therapy.

**ADMSCs**				
0.23 (−1.52, 1.92)	BMSCs			
1.06 (−0.76, 2.90)	0.84 (−0.30, 2.00)	NSCs		
3.74 (2.12, 5.27)	3.50 (2.84, 4.18)	2.66 (1.74, 3.58)	Placebo	
0.20 (−1.72, 2.09)	−0.03 (−1.42, 1.32)	−0.86 (−2.36, 0.58)	−3.53 (−4.72, −2.35)	UCMSCs

#### Eighth Week After Stem Cell Therapy

A total of 53 studies were included for network meta-analysis. The evidence network showed that there was no direct comparison between BMSCs vs. UCMSCs, BMSCs vs. ADMSCs, NSCs vs. UCMSCs, and NSCs vs. ADMSCs, and there were few studies on direct comparison between other types of stem cells. At the same time, the number of studies on BMSCs was the largest, see Figure 4D. Consistent with the results of traditional meta-analysis, the results of network meta-analysis indicated that rats had significantly higher BBB scores in stem cell groups compared to negative controls. However, the differences in BBB scores of rats between the four types of stem cells were not statistically significant, see Table 4. The comparison-correction funnel plot was asymmetric, suggesting that there may be publication bias and small sample effects, see Figure 5D. Rank ordering results showed that ADMSCs might be the most effective stem cells for SCI, see Supplementary Table S10.

**TABLE 4:** Network meta-analysis results 8 weeks after stem cell therapy.

ADMSCs				
1.01 (−1.28, 3.29)	BMSCs			
0.85 (−1.46, 3.22)	−0.15 (−1.30, 1.01)	NSCs		
4.25 (2.11, 6.44)	3.26 (2.48, 4.04)	3.40 (2.53, 4.28)	Placebo	
0.62 (−1.82, 3.07)	−0.38 (−1.75, 0.94)	−0.23 (−1.66, 1.15)	−3.64 (−4.76, −2.56)	UCMSCs

### Optimal Strategies for Stem Cell Therapy of SCI

Through a comprehensive analysis of the four types of stem cells, we found that UCMSCs may be the most effective stem cells for the treatment of SCI in the first week after stem cell transplantation, while ADMSCs may be the most effective stem cells in the third, fifth, and eighth weeks after stem cell transplantation. Based on the results of the comprehensive analysis at different time points, in addition, the limited sources of UCMSCs and the potential ethical concerns (for example, the umbilical cord is the genetic resource of pregnant women and newborns, which is protected by law, and the acceptance of umbilical cord varies greatly among people with diverse cultural backgrounds). We believe that ADMSCs have more therapeutic potential in SCI.

#### Optimal Dose of Stem Cell Transplantation

To avoid the influence of different transplantation routes and timings on the effect of different transplantation doses of stem cells, we performed a network meta-analysis in subgroups (for example, intralesional transplantation + acute phase transplantation, indicating that the subgroup is to explore the optimal therapeutic dose of stem cells undergoing intralesional transplantation during the acute phase). The third week after stem cell transplantation, (1) Intralesional transplantation + Acute phase transplantation: there was no significant difference in BBB scores between different doses [WMD = 1.84 (−6.21, 2.42)], ranking results showed that high dose transplantation had the best therapeutic effect (Possibility ranking: High dose = 84% > Low dose = 15%). (2) Intralesional transplantation + Subacute phase transplantation: there was no significant difference in BBB scores between different doses [WMD = 2.76 (−5.68, 10.98)], ranking results showed that high dose transplantation had the best therapeutic effect (Possibility ranking: High dose = 78% > Low dose = 21%). 3) Intravenous transplantation + Acute phase transplantation, Intravenous transplantation + Subacute phase transplantation: there were only high-dose study data. The fifth week after stem cell transplantation, (1) Intralesional transplantation + Acute phase transplantation: there was no significant difference in BBB scores between different doses [WMD = -0.55 (−3.17, 4.10)], ranking results showed that high dose transplantation had the best therapeutic effect (Possibility ranking: High dose = 65% > Low dose = 34%). (2) Intralesional transplantation + Subacute phase transplantation: there was no significant difference in BBB scores between different doses [WMD = 0.48 (−8.30, 9.44)], ranking results showed that high dose transplantation had the best therapeutic effect (Possibility ranking: High dose = 56% > Low dose = 44%). (3) Intravenous transplantation + Subacute phase transplantation: there were only high-dose study data. At the eighth week after stem cell transplantation, only a high dose of research data can be obtained.

In conclusion, the different subgroup analyses yielded consistent results that higher dose transplantation (≥1 × 10^6^) may have better therapeutic effects.

#### Optimal Route of Stem Cell Transplantation

To avoid the influence of different transplantation doses and timings on the effect of different transplantation routes of stem cells, we performed a network meta-analysis in subgroups. The third week after stem cell transplantation, (1) Acute phase transplantation + High dose transplantation: there was no significant difference in BBB scores between different routes [WMD = −2.99 (−9.70, 3.78)], ranking results showed that intralesional transplantation had the best therapeutic effect (Possibility ranking: intralesional transplantation = 84% > intravenous transplantation = 13%). (2) Subacute phase transplantation + High dose transplantation: there was no significant difference in BBB scores between different routes [WMD = 2.09 (−6.48, 10.92)], ranking results showed that intralesional transplantation had the best therapeutic effect (Possibility ranking: intralesional transplantation = 72% > intravenous transplantation = 28%). (3) Acute phase transplantation + Low dose transplantation, Subacute phase transplantation + Low dose transplantation: there were only data about intralesional transplantation. The fifth week after stem cell transplantation, (1) Acute phase transplantation + Low dose transplantation, Acute phase transplantation + High dose transplantation, Subacute phase transplantation + Low dose transplantation: there were only data about intralesional transplantation. (2) Subacute phase transplantation + High dose transplantation: there was no significant difference in BBB scores between different routes [WMD = 1.35 (−5.23, 7.43)], ranking results showed that intralesional transplantation had the best therapeutic effect (Possibility ranking: intralesional transplantation = 71% > intravenous transplantation = 29%). At the eighth week after stem cell transplantation, only the data of intralesional transplantation can be obtained.

In conclusion, the different subgroup analyses yielded consistent results that intralesional transplantation may have better therapeutic effects.

#### Optimal Timing of Stem Cell Transplantation

To avoid the influence of different transplantation doses and routes on the effect of different transplantation timings of stem cells, we performed a network meta-analysis in subgroups. The third week after stem cell transplantation, (1) Intralesional transplantation + Low dose transplantation: there was no significant difference in BBB scores between different timings [WMD = −0.06 (−4.17, 4.12)], ranking results showed that subacute phase transplantation had the best therapeutic effect (Possibility ranking: Subacute phase transplantation = 51% > Acute phase transplantation = 48%). (2) Intralesional transplantation + High dose transplantation: there was no significant difference in BBB scores between different timings [WMD = −4.64 (−16.60, 7.34)], ranking results showed that subacute phase transplantation had the best therapeutic effect (Possibility ranking: Subacute phase transplantation = 81% > Acute phase transplantation = 17%). (3) Intravenous transplantation + High dose transplantation: there was no significant difference in BBB scores between different timings [WMD = −0.38 (−7.56, 8.68)], ranking results showed that subacute phase transplantation had the best therapeutic effect (Possibility ranking: Subacute phase transplantation = 54% > Acute phase transplantation = 45%). The fifth week after stem cell transplantation, (1) Intralesional transplantation + Low dose transplantation: there was no significant difference in BBB scores between different timings [WMD = −3.05 (−4.92, 10.62)], ranking results showed that subacute phase transplantation had the best therapeutic effect (Possibility ranking: Subacute phase transplantation = 83% > Acute phase transplantation = 15%). (2) Intralesional transplantation + High dose transplantation: there was no significant difference in BBB scores between different timings [WMD = −3.02 (−11.93, 5.65)], ranking results showed that subacute phase transplantation had the best therapeutic effect (Possibility ranking: Subacute phase transplantation = 81% > Acute phase transplantation = 19%). (3) Intravenous transplantation + High dose transplantation: there were only study data about the subacute phase. The eighth week after stem cell transplantation, Intralesional transplantation + High dose transplantation: there was no significant difference in BBB scores between different timings [WMD = −4.48 (−12.11, 3.10)], ranking results showed that subacute phase transplantation had the best therapeutic effect (Possibility ranking: Subacute phase transplantation = 92% > Acute phase transplantation = 7%).

In conclusion, the different subgroup analyses yielded consistent results that subacute phase transplantation may have better therapeutic effects.

## Discussion

As a promising treatment, stem cells have made great progress in animal studies of SCI. However, the transformation of stem cells is an area that is particularly sensitive and needs to be treated with caution (Blight et al., 2009). Therefore, fully exploring the therapeutic potential and optimal therapeutic strategies of various stem cells in animal studies is critical to inform clinical practice and reduce the risk of clinical trials. Systematic review is an effective solution, which can strictly evaluate the real effect of stem cells in animal studies and the risk of their clinical transformation. Based on this, we comprehensively analyze the real therapeutic effects of stem cells in animal studies by traditional and network meta-analysis, while exploring the optimal stem cell treatment strategies.

### Summary of Evidence

#### Traditional Meta-Analysis

The results of traditional meta-analysis showed that rats in all sorts of stem cell groups exhibited better motor function scores after treatment compared with the negative control group. This is consistent with the results of other meta-analyses, which fully illustrates the great therapeutic potential of stem cells in SCI (Abbaszadeh et al., 2018). Although several types of stem cells can effectively promote the recovery of motor function in rats with SCI, there are currently many types of stem cells, and their routes, timings, and doses of transplantation are various. It is difficult to truly explore the real effect of stem cells under different treatment methods by comparing diverse types of stem cells with a negative control group only through traditional meta-analysis. The results obtained are also difficult to provide reference value for future animal and clinical studies.

In addition, stem cells have been preliminarily studied in the clinic. Unfortunately, they did not achieve the desired therapeutic effects. For example, studies of Muthu et al. have shown that stem cell therapy did not improve motor score and activities of daily living score in SCI patients (Muthu et al., 2020). Furthermore, there are still problems with the methodological quality of current clinical trials, such as the low number of patients recruited, non-randomized controlled studies, short follow-up time, and ethical issues, which reduce the credibility of the study (Goel, 2016). Also, it is difficult to conduct large-scale clinical trials to explore specific therapeutic strategies of stem cells. Thus, fully exploring the optimal therapeutic strategies for stem cells in preclinical studies is crucial.

#### Optimal Type of Stem Cell Transplantation

Previous studies have shown that mesenchymal stem cells have the advantages of diverse sources (bone marrow, fat, umbilical cord, amniotic fluid, etc.), relatively easy to obtain, few ethical issues, and expanded *in vitro* efficiency. In addition, the efficiency of migration of transplanted mesenchymal stem cells to lesion sites is higher than that of other types of stem cells (such as neural stem cells and adult stem cells), becoming the most promising stem cells for repairing SCI and the most studied stem cells in preclinical and clinical studies (Lu et al., 2006; Fischer et al., 2009; Dasari et al., 2014; Qu and Zhang, 2017). This is consistent with the results of our network meta-analysis that mesenchymal stem cells, especially BMSCs, are the focus of research. The initial study suggested that BMSCs were pluripotent and could be differentiated into nerve cells and glial cells. However, these views are being questioned. Transplanted cells may undergo trans-differentiation or fusion rather than differentiation (Kozorovitskiy and Gould, 2003). Therefore, the reliability of the therapeutic effect of BMSCs is questionable. Meanwhile, we further compared the effect of different stem cells in repairing SCI by network meta-analysis and found that the effect of BMSCs for repairing SCI was not the best one. We found that UCMSCs might be the most effective stem cells in the first week after transplantation, and the possible reason was that UCMSCs had lower immunogenicity and a lower incidence of immune rejection (Ryan et al., 2005). Instead, the results of 3, 5, and 8 weeks after transplantation showed that ADMSCs were the most effective stem cells. The possible reason is that ADMSCs could secrete more growth factors [such as BDNF, GDNF (Ohta et al., 2008)], regulate the activation of immune cells (Kim et al., 2015), neuroregeneration (Kolar et al., 2014; Menezes et al., 2014), anti-apoptosis (Kang et al., 2007), and multi-lineage differentiation (Kokai et al., 2014). Also, the activity of ADMSCs is three times higher than that of BMSCs (Han et al., 2015). In addition, ADMSCs senesce more slowly than other kinds of cells and, after several passages, can still differentiate into adipogenic, osteogenic, chondrogenic, myogenic, and neurogenic cells (Estes et al., 2010; Gimble et al., 2010; Meyerrose et al., 2010). However, ADMSCs is activated less rapidly than UCMSCs and, therefore, they do not exhibit better repair effects in the first week. However, different from autologous stem cells in clinic, 55 of the 188 studies included in the analysis were heterogeneous stem cells. The immune rejection caused by different sources of stem cells may affect or cover up the real effect of stem cells. Therefore, future studies need to further standardize the source of stem cells, so that the animal experiment process is as close as possible to clinical trials, to better promote the translation of animal experiment results into clinical practice.

Although stem cell therapy for SCI has made tremendous progress in animal studies and some clinical trials have been conducted, the reproducibility of stem cell therapy remains a great challenge (Ashammakhi et al., 2019). Standardizing the source, dose, timing, and route of transplantation of stem cells is an effective approach (Dominici et al., 2006). Thus, based on comprehensive analysis to determine that ADMSCs may be the most effective stem cells, we compared the efficacy of dose, route, and time of stem cell transplantation at different time points by network meta-analysis.

#### Optimal Dose of Stem Cell Transplantation

The results of meta-analysis based on a small number of clinical trials showed that there was no statistical difference between the scores for motor function of patients who received low-dose (<5 × 10^7^) and high-dose (>5 × 10^7^) cell transplantation. Although the authors did not come up with inconsistent results between different doses, they were cautious that the dose of stem cell transplantation remained a key factor in determining transplant outcomes and needed further study (Muthu et al., 2020). Unlike clinical studies, stem cell therapy involves a larger range of transplantation doses (1 × 10^4^–1 × 10^8^) in animal studies, and the same dose does not necessarily show the same effect in animals and humans. In view of the great difficulty comparing different cell transplantation doses in clinical practice, it is especially important to determine the optimal transplantation treatment of stem cells in animal studies to guide clinical practice. We subjected studies with different doses of transplantation to a network meta-analysis, which showed that high-dose transplantation of stem cell (≥1 × 10^6^) was superior to low-dose transplantation (≤1 × 10^6^) in promoting motor function recovery. Although the division of transplantation doses in our study was different from that of Hosseini et al. (doses were divided into ≥3 × 10^6^ and <3 × 10^6^), the results both showed that higher doses of stem cell transplantation may be more effective (Hosseini et al., 2015). In addition, Hosseini et al. combined the results of different genera (rats, mice, and dogs), different motor function score scales (e.g., BBB scale, BMS score, and Olby test), which led to great heterogeneity and affected the reliability of their research results. Only BBB scores of rats were combined for analysis in our study to reduce the heterogeneity among different studies. Therefore, the optimal dose of stem cell transplantation that we derived is more precise and reliable. It is worth noting that Kumamaru et al. believed high-dose cell transplantation would trigger a strong inflammatory response that negatively affects the recovery of motor function (Kumamaru et al., 2012). However, due to the limited number of current studies, it is not possible to further clarify a specific transplantation dose. Also, limited to the significant differences that exist in animals and humans, the effective transplantation dose for animals with SCI may not be applicable in the clinic. Therefore, future studies should further compare the differences in the effects of the same or different transplantation doses in animals and clinical patients to determine the applicability of animal experimental results for clinical patients. Also, further determination of the optimal dose of transplantation is necessary to maximize the likelihood of achieving motor function regeneration.

#### Optimal Route of Stem Cell Transplantation

The location of stem cell transplantation after SCI impacts transplanted cell fate (Assinck et al., 2017). Since the first transplantation of mesenchymal stem cells in the 1990s, intravenous administration has attracted much attention because it does not require imaging equipment guidance and surgical operation. In addition, 36 clinical trials involving 1012 patients have not found any complications, such as acute transfusion toxicity, fever, organ system complications, infection, and longer-term adverse events (death, malignancy) related to intravenous injection of mesenchymal stem cells (Lalu et al., 2012). However, mesenchymal stem cells should have migrated through the cerebrospinal fluid barrier to the diseased spinal cord under the action of chemokines (Cofano et al., 2019). The actual situation is that most of the transplanted cells are trapped in the lung through intravenous transplantation, and only a small part of the cells migrates to the lesion site. Therefore, the transplanted stem cells cannot fully play the role of repair due to the limited number of actual cells reaching the lesion site (Liau et al., 2020). Direct transplantation of stem cells to the site of damage suggested higher transplant efficiency and enhanced repair by Vaquero et al. (2006) and Shin et al. (2013). This is consistent with the results of our network meta-analysis showing that intralesional transplantation performed better than with intravenous transplantation. It is worth noting that the modality of intralesional transplantation has shown superior results in rats because the experimental personnel directly transplanted cells to the injured site after animal modeling. However, there is usually no open injury in clinical patients with SCI. Intralesional transplantation may lead to secondary SCI and infection. Thus, whether intralesional transplantation is appropriate for humans remains controversial. In addition, intralesional transplantation also faces great challenges in animal studies, mainly that transplantation may lead to further SCI (e.g., needle stick injury), which, combined with the uncontrollability of the dose, speed, pressure, etc., of transplantation, can easily aggravate SCI. Currently, intrathecal, subarachnoid, and ventricle have gradually gained attention as potential routes of transplantation, but the related studies are few and difficult to illustrate the problems. In summary, the goal of different transplantation routes is to increase the efficiency of stem cell colonization to the injury site in order to exert repair effects. Thus, future animal studies should further explore the advantages and disadvantages of different routes and the effects of specific operation on the recovery of motor function in SCI animals.

#### Optimal Timing of Stem Cell Transplantation

The most important factor affecting the therapeutic effect of patients with SCI is the time when stem cells are transplanted to the injured site to play their targeted role (Chhabra and Sarda, 2017a). At present, there is no clear consensus on the timing of transplantation, but studies have shown that longer intervals to transplantation are associated with more severe SCI (Oyinbo, 2011). Also, the meta-analysis by Hosseini et al. argued that stem cell transplantation in the acute phase (first 4 days) after SCI was more effective than that in the subacute phase (4 days later) (Hosseini et al., 2015). However, the results of our network meta-analysis for different time points all showed that transplantation in the subacute phase (3–14 days) performed better than transplantation in the acute phase (≤3 days). The discrepancy in our findings with those of Hosseini et al., may be explained by differences in the division of the period of SCI and the fact that the transplantations in our study were ADMSCs and those in Hosseini et al. were BMSCs. Although neither our studies nor Hosseini et al. have provided a reasonable basis for dividing the period of SCI, previously published animal studies suggested that the cytotoxic environment caused by inflammatory response in acute rats affects the survival and differentiation of stem cells (Mothe and Tator, 2013). However, glial scar formation is slower in the subacute phase, the inflammatory response subsides, and transplanted stem cells can fully exert the repair effect (Jeong et al., 2020). At the same time, based on actual clinical conditions, most patients of SCI occur outside the hospital, and bringing patients to the hospital for first aid can take hours, as well as time is needed for the preparation and evaluation of cellular products. Clinical patients cannot get stem cell transplantation immediately after SCI as animals can. Thus, the results of cell transplantation during the acute phase need to be interpreted and used with caution. In conclusion, we believe that cell transplantation in the subacute phase is more effective and more suitable for clinical practice. Moreover, although many studies suggest that the effect of chronic transplantation is poor, it is because of the loss of plasticity of nerve cells in the injured site and the formation of extensive scars and cysts that do not have therapeutic effects (Chhabra and Sarda, 2017b). However, the included studies rarely transplanted after 14 days of SCI, and therefore, we could not evaluate the effect of transplantation in the chronic phase. Future studies should further explore the effects of transplantation in the chronic phase.

In summary, through a comprehensive analysis of the 188 studies included, we found that all 15 kinds of stem cells could significantly promote the recovery of motor function in rats with SCI. By network meta-analysis, we found that ADMSCs had the greatest therapeutic potential for SCI. Moreover, higher doses of stem cell transplantation were more effective, transplantation in the subacute phase was the optimal timing and intralesional transplantation was the optimal route. However, the therapeutic effects of the remaining 11 kinds of stem cells that were not included in the analysis still need more animal studies to explore them.

### Quality of Evidence

Based on strict systematic review, we found that the evidence quality of animal experiments was not high, which reduced the reliability of the experimental results to a certain extent and increased the risk of animal experimental results transforming to clinical practice. The possible reasons included:

1) There was heterogeneity in the included studies. Although 21 items clearly illustrate the use of BBB score, the difference in the familiarity and understanding of BBB scoring criteria among different experimental researchers will lead to differences in the scores obtained from different studies on the same degree of SCI and ultimately affect the reliability of the results (Tator, 2006). Therefore, the reliability of our data based on this subjective score is greatly affected by the scorer. This is the biggest factor affecting the reliability of our conclusions. Moreover, sources are different in the same stem cell, which include autologous, allogeneic, xenograft, differences in modeling of SCI (impactor shape, impact weight, impact height), surgical details (surgical procedure, time, blood loss), postoperative animal care, etc., which can lead to some heterogeneity between studies.
2) Insufficient intrinsic authenticity of included studies. Of the 188 included studies, there were 166 RCTs. However, none of the 153 studies clearly reported the method of randomization. While the remaining 13 studies all reported specific methods of randomization, none of them reported whether to implement covert grouping, resulting in high possibility of selective bias. Therefore, future experiments should be strictly randomized and covertly grouped to reduce selectivity bias in animal experiments. Moreover, all included studies did not report blinding of caregivers/researchers or outcome assessors. Although there was no need to blind animals, the researchers in most studies were animal breeders, who might introduce implementation bias and measurement bias due to subjective factors in the process of intervention, result measurement, and evaluation. Therefore, it is necessary to be blind in the stage of intervention implementation and outcome measurement to reduce the implementation bias and measurement bias during the experiment and increase the authenticity of the experimental results (Zeng et al., 2013; Tao et al., 2019). Moreover, for the determination of outcome indicators, in addition to the implementation of effective scientific blind method can avoid the impact of measurement bias on the measurement results, the qualification of the measurement, the consistency of the measurement on different animals, the accuracy and scientificity of the validity standards will affect the measurement of the results to different degrees (Sessler and Imrey, 2015). However, none of the 188 studies included in our study reported the qualification of the surveyors and their standards and specific measurement process at the time of the outcome measurement. Therefore, future studies should comprehensively report their specific experimental implementation details in order to improve the reproducibility and reliability of animal experimental results (Dell et al., 2002). None of the included studies had access to their protocol, and it was not possible to make a final judgment on whether all their results were reported as planned and unbiased. The selective reporting of animal experiments may lead to the generation of publication bias, which affects the reliability of conclusions of systematic review, and even draws the opposite conclusions (Korevaar et al., 2011). Therefore, prospective registration of animal experiments for access to their raw data may be considered at the level of industry associations, countries, etc. In addition, it is very necessary that animal studies in the future provide original data as online appendices to improve transparency of the whole process and improve the quality of animal experiments (Ritskes-Hoitinga et al., 2014).
3) Insufficient external authenticity of included studies: External authenticity refers to the extent to which the clinical results can be repeated in the target population and daily population (Wu et al., 2011). The external authenticity of several aspects should be considered when translating animal experimental results to clinical trials: 1) Many studies have been studied using immunodeficient animal models that vary greatly in immune responses to SCI. Although this change in the immune system can overcome rejection of transplanted cells, SCI models with immunodeficiency may not accurately simulate human conditions (Kobayashi et al., 2012). 2) The patient’s medical history, and internal or external physical conditions may affect the efficacy of stem cells. Aging and diabetes, for example, result in impaired proliferation of stem cells, decreased angiogenic capacity, and reduced wound healing (El-Ftesi et al., 2009). In addition, animal experiments have difficulty in simulating multiple body conditions in humans simultaneously. 3) In animal studies, the efficacy of stem cells can only be explored by relatively objective outcome measures such as BBB scores, inflammatory factors, and so on, whereas subtle changes in sensory function, nerve root movement, and pain that are of clinical concern cannot be fully investigated by animal experiments. 4) SCI occurs in the cervical spine in more than 60% of patients (Dvorak et al., 2014), however, preclinical studies have used lumbar and thoracic models that are technically easier to implement, reducing the applicability of animal findings in clinical studies (Tator, 2006; Vismara et al., 2017). Therefore, future studies should consider the development of more clinically representative SCI models. 5) The stem cells used clinically are all human-derived stem cells, and the diverse sources of stem cells in animal studies can lead to strong immune responses, which affects the authenticity of the results of animal studies (Drukker and Benvenisty, 2004). 6) The characteristics of accelerating tumor growth have raised concerns among clinicians and patients, which is also one of the key factors in their susceptibility to ethical problems (Volarevic et al., 2018). However, animal studies have a short follow-up time, and little attention has been paid to the tumorigenic effect of stem cells. 7) Longer follow-up results in a more comprehensive prediction of the trajectory of motor function recovery in SCI animals, which may reduce the number of subjects required for subsequent clinical trials and better guide clinical practice. However, few preclinical studies have extended the follow-up to 2 months after cell transplantation. Therefore, future animal studies should extend the follow-up time to further observe the therapeutic effects of stem cells (Assinck et al., 2017). Because of the aforementioned limitations of external authenticity, it has resulted in difficulty to have enough evidence to support the results of the animal experiments included in this study to enable further clinical trials.

### Strengths and Limitations of the Present Study

Key strengths of this systematic review: (1) Based on animal studies, the real effects and limitations of stem cells for SCI were systematically evaluated and analyzed, while the existing problems and directions for improvement in the current field were pointed out. (2) The results of stem cell repair of SCI were analyzed at different time points, and the effect of stem cells in the whole treatment process was studied more comprehensively. (3) Based on network meta-analysis, which comprehensively compared the real effects of different kinds of stem cells for repairing SCI, the optimal strategy of the stem cell was derived, which was the first time in the current field. (4) We conducted the network meta-analysis in subgroups to avoid the interference of different transplantation doses, timings, and routes of stem cells. (5) Based on the internationally recognized SYRCLE bias risk assessment tool, the internal bias risk of animal studies was strictly evaluated, and the problems in the design and implementation of animal studies in this field were pointed out. At the same time, suggestions on how to improve the quality of animal experiments were given.

Limitations of this systematic review: (1) Although there is a certain basis for data selection based on the recovery of motor function and inflammatory response in SCI rats. However, whether the data selection method was reliable was still uncertain. 2) Four types of stem cells selectively were included for network meta-analysis, which may overlook the potential therapeutic role of other kinds of stem cells. 3) We could not accurately identify the source of heterogeneity. Therefore, we adopted a random effects model for meta-analysis, making our conclusions more conservative. 4) Only Chinese and English databases were retrieved, which may lead to a certain language bias. 5) Grey literature and conference abstracts were not searched, potentially leading to the generation of publication bias.

## Conclusion

Through traditional meta-analysis of 15 different types of stem cells, we found that all sorts of stem cells exhibited great therapeutic potential in preclinical studies of spinal cord injury compared to a negative group. Based on the network meta-analysis, we found that adipose-derived mesenchymal stem cells (ADMSCs) have the greatest therapeutic potential for SCI. Moreover, a higher dose (≥1 × 10^6^) of stem cell transplantation had better therapeutic effect, transplantation in the subacute phase was the optimal timing and intralesional transplantation was the optimal route. The remaining 11 kinds of stem cells were not included in network meta-analysis because of the small sample bias due to the small number of studies and being easily affected by the results of a single study. But they still have a great therapeutic potential compared with placebo and need more high-quality animal studies to explore in the future.

As the basis for the design and implementation of subsequent early clinical trials, the quality of preclinical studies directly determines whether the research results can be transformed into clinical practice. Through a comprehensive analysis of included studies, we considered current animal studies with stem cells to repair SCI still have certain problems regarding randomization, allocation concealment, blinding, and measurement and reporting of outcomes. Especially for BBB scores that rely on subjective evaluation, these problems can severely reduce the quality of animal studies. Therefore, future studies need to further standardize the implementation and reporting of animal studies to improve the quality of evidence from preclinical studies and reduce the risk of translation of preclinical findings to the clinic.

## Data Availability

The original contributions presented in the study are included in the article/Supplementary Material, further inquiries can be directed to the corresponding authors.

## References

[B1] Abbaszadeh H. A. Niknazar S. Darabi S. Ahmady Roozbahany N. Noori-Zadeh A. Ghoreishi S. K. (2018). Stem Cell Transplantation and Functional Recovery after Spinal Cord Injury: a Systematic Review and Meta-Analysis. Anat. Cel Biol 51, 180–188. 10.5115/acb.2018.51.3.180 PMC6172584 30310710

[B2] Abematsu M. Tsujimura K. Yamano M. Saito M. Kohno K. Kohyama J. (2010). Neurons Derived from Transplanted Neural Stem Cells Restore Disrupted Neuronal Circuitry in a Mouse Model of Spinal Cord Injury. J. Clin. Invest. 120, 3255–3266. 10.1172/JCI42957 20714104 PMC2929730

[B3] Amemori T. Romanyuk N. Jendelova P. Herynek V. Turnovcova K. Prochazka P. (2013). Human Conditionally Immortalized Neural Stem Cells Improve Locomotor Function after Spinal Cord Injury in the Rat. Stem Cel Res Ther 4, 68. 10.1186/scrt219 PMC3706805 23759119

[B4] Ashammakhi N. Kim H. J. Ehsanipour A. Bierman R. D. Kaarela O. Xue C. (2019). Regenerative Therapies for Spinal Cord Injury. Tissue Eng. Part. B Rev. 25, 471–491. 10.1089/ten.TEB.2019.0182 31452463 PMC6919264

[B5] Assinck P. Duncan G. J. Hilton B. J. Plemel J. R. Tetzlaff W. (2017). Cell Transplantation Therapy for Spinal Cord Injury. Nat. Neurosci. 20, 637–647. 10.1038/nn.4541 28440805

[B6] Barnabé-Heider F. Frisén J. (2008). Stem Cells for Spinal Cord Repair. Cell stem cell 3, 16–24. 10.1016/j.stem.2008.06.011 18593555

[B7] Basso D. M. Beattie M. S. Bresnahan J. C. Anderson D. K. Faden A. I. Gruner J. A. (1996). MASCIS Evaluation of Open Field Locomotor Scores: Effects of Experience and Teamwork on Reliability. Multicenter Animal Spinal Cord Injury Study. J. Neurotrauma 13, 343–359. 10.1089/neu.1996.13.343 8863191

[B8] Beck K. D. Nguyen H. X. Galvan M. D. Salazar D. L. Woodruff T. M. Anderson A. J. (2010). Quantitative Analysis of Cellular Inflammation after Traumatic Spinal Cord Injury: Evidence for a Multiphasic Inflammatory Response in the Acute to Chronic Environment. Brain 133, 433–447. 10.1093/brain/awp322 20085927 PMC2858013

[B9] Blight A. Curt A. Ditunno J. F. Dobkin B. Ellaway P. Fawcett J. (2009). Position Statement on the Sale of Unproven Cellular Therapies for Spinal Cord Injury: the International Campaign for Cures of Spinal Cord Injury Paralysis. Spinal cord 47, 713–714. 10.1038/sc.2008.179 19417762

[B10] Chhabra H. S. Sarda K. (2017). Clinical Translation of Stem Cell Based Interventions for Spinal Cord Injury - Are We There yet. Adv. Drug Deliv. Rev. 120, 41–49. 10.1016/j.addr.2017.09.021 28964881

[B11] Chhabra H. S. Sarda K. (2017). Clinical Translation of Stem Cell Based Interventions for Spinal Cord Injury - Are We There yet. Adv. Drug Deliv. Rev. 120, 41–49. 10.1016/j.addr.2017.09.021 28964881

[B12] Cofano F. Boido M. Monticelli M. Zenga F. Ducati A. Vercelli A. (2019). Mesenchymal Stem Cells for Spinal Cord Injury: Current Options, Limitations, and Future of Cell Therapy. Int. J. Mol. Sci. 20. 10.3390/ijms20112698 PMC6600381 31159345

[B13] Cox C. S. Jr (2018). Cellular Therapy for Traumatic Neurological Injury. Pediatr. Res. 83, 325–332. 10.1038/pr.2017.253 28985200

[B14] Dasari V. R. Veeravalli K. K. Dinh D. H. (2014). Mesenchymal Stem Cells in the Treatment of Spinal Cord Injuries: A Review. World J. Stem Cell 6, 120–133. 10.4252/wjsc.v6.i2.120 PMC3999770 24772239

[B15] De Feo D. Merlini A. Laterza C. Martino G. (2012). Neural Stem Cell Transplantation in central Nervous System Disorders: from Cell Replacement to Neuroprotection. Curr. Opin. Neurol. 25, 322–333. 10.1097/WCO.0b013e328352ec45 22547103

[B16] Dell R. B. Holleran S. Ramakrishnan R. (2002). Sample Size Determination. ILAR J. 43, 207–213. 10.1093/ilar.43.4.207 12391396 PMC3275906

[B17] Dominici M. Le Blanc K. Mueller I. Slaper-Cortenbach I. Marini F. Krause D. (2006). Minimal Criteria for Defining Multipotent Mesenchymal Stromal Cells. The International Society for Cellular Therapy Position Statement. Cytotherapy 8, 315–317. 10.1080/14653240600855905 16923606

[B18] Drukker M. Benvenisty N. (2004). The Immunogenicity of Human Embryonic Stem-Derived Cells. Trends Biotechnol. 22, 136–141. 10.1016/j.tibtech.2004.01.003 15036864

[B19] Dvorak M. F. Noonan V. K. Fallah N. Fisher C. G. Rivers C. S. Ahn H. (2014). Minimizing Errors in Acute Traumatic Spinal Cord Injury Trials by Acknowledging the Heterogeneity of Spinal Cord Anatomy and Injury Severity: an Observational Canadian Cohort Analysis. J. Neurotrauma 31, 1540–1547. 10.1089/neu.2013.3278 24811484 PMC4161054

[B20] El-Ftesi S. Chang E. I. Longaker M. T. Gurtner G. C. (2009). Aging and Diabetes Impair the Neovascular Potential of Adipose-Derived Stromal Cells. Plast. Reconstr. Surg. 123, 475–485. 10.1097/PRS.0b013e3181954d08 19182604 PMC2878769

[B21] Estes B. T. Diekman B. O. Gimble J. M. Guilak F. (2010). Isolation of Adipose-Derived Stem Cells and Their Induction to a Chondrogenic Phenotype. Nat. Protoc. 5, 1294–1311. 10.1038/nprot.2010.81 20595958 PMC3219531

[B22] Fischer U. M. Harting M. T. Jimenez F. Monzon-Posadas W. O. Xue H. Savitz S. I. (2009). Pulmonary Passage Is a Major Obstacle for Intravenous Stem Cell Delivery: the Pulmonary First-Pass Effect. Stem Cell Dev 18, 683–692. 10.1089/scd.2008.0253 PMC3190292 19099374

[B23] Gimble J. M. Guilak F. Bunnell B. A. (2010). Clinical and Preclinical Translation of Cell-Based Therapies Using Adipose Tissue-Derived Cells. Stem Cel Res Ther 1, 19. 10.1186/scrt19 PMC2905095 20587076

[B24] Goel A. (2016). Stem Cell Therapy in Spinal Cord Injury: Hollow Promise or Promising Science. J. Craniovertebr Junction Spine 7, 121–126. 10.4103/0974-8237.181880 27217662 PMC4872563

[B25] Guest J. D. Hiester E. D. Bunge R. P. (2005). Demyelination and Schwann Cell Responses Adjacent to Injury Epicenter Cavities Following Chronic Human Spinal Cord Injury. Exp. Neurol. 192, 384–393. 10.1016/j.expneurol.2004.11.033 15755556

[B26] Guo S. Wang L. Xie Y. Luo X. Zhang S. Xiong L. (2019). Bibliometric and Visualized Analysis of Stem Cells Therapy for Spinal Cord Injury Based on Web of Science and CiteSpace in the Last 20 Years. World Neurosurg. 132, e246–e258. 10.1016/j.wneu.2019.08.191 31493613

[B27] Han S. Sun H. M. Hwang K. C. Kim S. W. (2015). Adipose-derived Stromal Vascular Fraction Cells: Update on Clinical Utility and Efficacy. Crit. Rev. Eukaryot. Gene Expr. 25, 145–152. 10.1615/critreveukaryotgeneexpr.2015013057 26080608

[B28] Hooijmans C. R. Rovers M. M. de Vries R. B. Leenaars M. Ritskes-Hoitinga M. Langendam M. W. (2014). SYRCLE's Risk of Bias Tool for Animal Studies. BMC Med. Res. Methodol. 14, 43. 10.1186/1471-2288-14-43 24667063 PMC4230647

[B29] Hosseini M. Yousefifard M. Aziznejad H. Nasirinezhad F. (2015). The Effect of Bone Marrow-Derived Mesenchymal Stem Cell Transplantation on Allodynia and Hyperalgesia in Neuropathic Animals: A Systematic Review with Meta-Analysis. Biol. Blood Marrow Transpl. 21, 1537–1544. 10.1016/j.bbmt.2015.05.008 25985918

[B30] Hu X. C. Lu Y. B. Yang Y. N. Kang X. W. Wang Y. G. Ma B. (2021). Progress in Clinical Trials of Cell Transplantation for the Treatment of Spinal Cord Injury: How many Questions Remain Unanswered. Neural Regen. Res. 16, 405–413. 10.4103/1673-5374.293130 32985458 PMC7996007

[B31] Huang L. Fu C. Xiong F. He C. Wei Q. (2021). Stem Cell Therapy for Spinal Cord Injury. Cel Transpl. 30, 963689721989266. 10.1177/0963689721989266 PMC7876757 33559479

[B32] Jeong S. K. Choi I. Jeon S. R. (2020). Current Status and Future Strategies to Treat Spinal Cord Injury with Adult Stem Cells. J. Korean Neurosurg. Soc. 63, 153–162. 10.3340/jkns.2019.0146 31805758 PMC7054109

[B33] Kaner T. Karadag T. Cirak B. Erken H. A. Karabulut A. Kiroglu Y. (2010). The Effects of Human Umbilical Cord Blood Transplantation in Rats with Experimentally Induced Spinal Cord Injury. J. Neurosurg. Spine 13, 543–551. 10.3171/2010.4.SPINE09685 20887153

[B34] Kang S. K. Yeo J. E. Kang K. S. Phinney D. G. (2007). Cytoplasmic Extracts from Adipose Tissue Stromal Cells Alleviates Secondary Damage by Modulating Apoptosis and Promotes Functional Recovery Following Spinal Cord Injury. Brain Pathol. 17, 263–275. 10.1111/j.1750-3639.2007.00070.x 17465991 PMC8095508

[B35] Karamouzian S. Nematollahi-Mahani S. N. Nakhaee N. Eskandary H. (2012). Clinical Safety and Primary Efficacy of Bone Marrow Mesenchymal Cell Transplantation in Subacute Spinal Cord Injured Patients. Clin. Neurol. Neurosurg. 114, 935–939. 10.1016/j.clineuro.2012.02.003 22464434

[B36] Kim Y. Jo S. H. Kim W. H. Kweon O. K. (2015). Antioxidant and Anti-inflammatory Effects of Intravenously Injected Adipose Derived Mesenchymal Stem Cells in Dogs with Acute Spinal Cord Injury. Stem Cel Res Ther 6, 229. 10.1186/s13287-015-0236-5 PMC4660672 26612085

[B37] Kjell J. Olson L. (2016). Rat Models of Spinal Cord Injury: from Pathology to Potential Therapies. Dis. Model. Mech. 9, 1125–1137. 10.1242/dmm.025833 27736748 PMC5087825

[B38] Kobayashi Y. Okada Y. Itakura G. Iwai H. Nishimura S. Yasuda A. (2012). Pre-evaluated Safe Human iPSC-Derived Neural Stem Cells Promote Functional Recovery after Spinal Cord Injury in Common Marmoset without Tumorigenicity. PloS one 7, e52787. e52787. 10.1371/journal.pone.0052787 23300777 PMC3531369

[B39] Kokai L. E. Marra K. Rubin J. P. (2014). Adipose Stem Cells: Biology and Clinical Applications for Tissue Repair and Regeneration. Transl Res. 163, 399–408. 10.1016/j.trsl.2013.11.009 24361334

[B40] Kolar M. K. Kingham P. J. Novikova L. N. Wiberg M. Novikov L. N. (2014). The Therapeutic Effects of Human Adipose-Derived Stem Cells in a Rat Cervical Spinal Cord Injury Model. Stem Cell Dev 23, 1659–1674. 10.1089/scd.2013.0416 24803143

[B41] Kooijmans H. Post M. W. M. Stam H. J. van der Woude L. H. V. Spijkerman D. C. M. Snoek G. J. (2017). Effectiveness of a Self-Management Intervention to Promote an Active Lifestyle in Persons with Long-Term Spinal Cord Injury: The HABITS Randomized Clinical Trial. Neurorehabil. Neural Repair 31, 991–1004. 10.1177/1545968317736819 29256337

[B42] Korevaar D. A. Hooft L. ter Riet G. (2011). Systematic Reviews and Meta-Analyses of Preclinical Studies: Publication Bias in Laboratory Animal Experiments. Lab. Anim. 45, 225–230. 10.1258/la.2011.010121 21737463

[B43] Kozorovitskiy Y. Gould E. (2003). Stem Cell Fusion in the Brain. Nat. Cel Biol 5, 952–954. 10.1038/ncb1103-952 14593417

[B44] Kumamaru H. Ohkawa Y. Saiwai H. Yamada H. Kubota K. Kobayakawa K. (2012). Direct Isolation and RNA-Seq Reveal Environment-dependent Properties of Engrafted Neural Stem/progenitor Cells. Nat. Commun. 3, 1140. 10.1038/ncomms2132 23072808

[B45] Lalu M. M. McIntyre L. Pugliese C. Fergusson D. Winston B. W. Marshall J. C. (2012). Safety of Cell Therapy with Mesenchymal Stromal Cells (SafeCell): a Systematic Review and Meta-Analysis of Clinical Trials. PloS one 7, e47559. e47559. 10.1371/journal.pone.0047559 23133515 PMC3485008

[B46] Liau L. L. Looi Q. H. Chia W. C. Subramaniam T. Ng M. H. Law J. X. (2020). Treatment of Spinal Cord Injury with Mesenchymal Stem Cells. Cell Biosci 10, 112. 10.1186/s13578-020-00475-3 32983406 PMC7510077

[B47] Lu L. L. Liu Y. J. Yang S. G. Zhao Q. J. Wang X. Gong W. (2006). Isolation and Characterization of Human Umbilical Cord Mesenchymal Stem Cells with Hematopoiesis-Supportive Function and Other Potentials. Haematologica 91, 1017–1026. 16870554

[B48] Menezes K. Nascimento M. A. Gonçalves J. P. Cruz A. S. Lopes D. V. Curzio B. (2014). Human Mesenchymal Cells from Adipose Tissue deposit Laminin and Promote Regeneration of Injured Spinal Cord in Rats. PloS one 9, e96020. e96020. 10.1371/journal.pone.0096020 24830794 PMC4022508

[B49] Meyerrose T. Olson S. Pontow S. Kalomoiris S. Jung Y. Annett G. (2010). Mesenchymal Stem Cells for the Sustained *In Vivo* Delivery of Bioactive Factors. Adv. Drug Deliv. Rev. 62, 1167–1174. 10.1016/j.addr.2010.09.013 20920540 PMC3815452

[B50] Miron V. E. Boyd A. Zhao J. W. Yuen T. J. Ruckh J. M. Shadrach J. L. (2013). M2 Microglia and Macrophages Drive Oligodendrocyte Differentiation during CNS Remyelination. Nat. Neurosci. 16, 1211–1218. 10.1038/nn.3469 23872599 PMC3977045

[B51] Morita T. Sasaki M. Kataoka-Sasaki Y. Nakazaki M. Nagahama H. Oka S. (2016). Intravenous Infusion of Mesenchymal Stem Cells Promotes Functional Recovery in a Model of Chronic Spinal Cord Injury. Neuroscience 335, 221–231. 10.1016/j.neuroscience.2016.08.037 27586052

[B52] Mosley M. C. Lim H. J. Chen J. Yang Y. H. Li S. Liu Y. (2017). Neurite Extension and Neuronal Differentiation of Human Induced Pluripotent Stem Cell Derived Neural Stem Cells on Polyethylene Glycol Hydrogels Containing a Continuous Young's Modulus Gradient. J. Biomed. Mater. Res. A. 105, 824–833. 10.1002/jbm.a.35955 27798956

[B53] Mothe A. J. Tator C. H. (2012). Advances in Stem Cell Therapy for Spinal Cord Injury. J. Clin. Invest. 122, 3824–3834. 10.1172/JCI64124 23114605 PMC3484454

[B54] Mothe A. J. Tator C. H. (2013). Review of Transplantation of Neural Stem/progenitor Cells for Spinal Cord Injury. Int. J. Dev. Neurosci. 31, 701–713. 10.1016/j.ijdevneu.2013.07.004 23928260

[B55] Muthu S. Jeyaraman M. Gulati A. Arora A. (2020). Current Evidence on Mesenchymal Stem Cell Therapy for Traumatic Spinal Cord Injury: Systematic Review and Meta-Analysis. Cytotherapy 23 (3), 186–197. 10.1016/j.jcyt.2020.09.007 33183980

[B56] Nagoshi N. Okano H. (2017). Applications of Induced Pluripotent Stem Cell Technologies in Spinal Cord Injury. J. Neurochem. 141, 848–860. 10.1111/jnc.13986 28199003

[B57] Nakajima H. Uchida K. Guerrero A. R. Watanabe S. Sugita D. Takeura N. (2012). Transplantation of Mesenchymal Stem Cells Promotes an Alternative Pathway of Macrophage Activation and Functional Recovery after Spinal Cord Injury. J. Neurotrauma 29, 1614–1625. 10.1089/neu.2011.2109 22233298 PMC3353766

[B58] Nutt S. E. Chang E. A. Suhr S. T. Schlosser L. O. Mondello S. E. Moritz C. T. (2013). Caudalized Human iPSC-Derived Neural Progenitor Cells Produce Neurons and Glia but Fail to Restore Function in an Early Chronic Spinal Cord Injury Model. Exp. Neurol. 248, 491–503. 10.1016/j.expneurol.2013.07.010 23891888 PMC4109283

[B59] Ohta Y. Takenaga M. Tokura Y. Hamaguchi A. Matsumoto T. Kano K. (2008). Mature Adipocyte-Derived Cells, Dedifferentiated Fat Cells (DFAT), Promoted Functional Recovery from Spinal Cord Injury-Induced Motor Dysfunction in Rats. Cel Transpl. 17, 877–886. 10.3727/096368908786576516 19069631

[B60] Oliveri R. S. Bello S. Biering-Sørensen F. (2014). Mesenchymal Stem Cells Improve Locomotor Recovery in Traumatic Spinal Cord Injury: Systematic Review with Meta-Analyses of Rat Models. Neurobiol. Dis. 62, 338–353. 10.1016/j.nbd.2013.10.014 24148857

[B61] Organization W. H. Society I. S. C. (2013). International Perspectives on Spinal Cord Injury. World Health Organization.

[B62] Osaka M. Honmou O. Murakami T. Nonaka T. Houkin K. Hamada H. (2010). Intravenous Administration of Mesenchymal Stem Cells Derived from Bone Marrow after Contusive Spinal Cord Injury Improves Functional Outcome. Brain Res. 1343, 226–235. 10.1016/j.brainres.2010.05.011 20470759

[B63] Oyinbo C. A. (2011). Secondary Injury Mechanisms in Traumatic Spinal Cord Injury: a Nugget of This Multiply cascade. Acta Neurobiol. Exp. (Wars) 71, 281–299. 21731081 10.55782/ane-2011-1848

[B64] Qu J. Zhang H. (2017). Roles of Mesenchymal Stem Cells in Spinal Cord Injury. Stem Cell Int 2017, 5251313. 10.1155/2017/5251313 PMC5467343 28630630

[B65] Ritskes-Hoitinga M. Leenaars M. Avey M. Rovers M. Scholten R. (2014). Systematic Reviews of Preclinical Animal Studies Can Make Significant Contributions to Health Care and More Transparent Translational Medicine. Cochrane database Syst. Rev. 28, Ed000078. 10.1002/14651858.ed000078 PMC10845857 24719910

[B66] Rowland J. W. Hawryluk G. W. Kwon B. Fehlings M. G. (2008). Current Status of Acute Spinal Cord Injury Pathophysiology and Emerging Therapies: Promise on the Horizon. Neurosurg. Focus 25, E2. 10.3171/FOC.2008.25.11.E2 18980476

[B67] Ryan J. M. Barry F. P. Murphy J. M. Mahon B. P. (2005). Mesenchymal Stem Cells Avoid Allogeneic Rejection. J. Inflamm. (Lond) 2, 8. 10.1186/1476-9255-2-8 16045800 PMC1215510

[B68] Sessler D. I. Imrey P. B. (2015). Clinical Research Methodology 3: Randomized Controlled Trials. Anesth. Analg 121, 1052–1064. 10.1213/ANE.0000000000000862 26378705

[B69] Shin D. A. Kim J. M. Kim H. I. Yi S. Ha Y. Yoon D. H. (2013). Comparison of Functional and Histological Outcomes after Intralesional, Intracisternal, and Intravenous Transplantation of Human Bone Marrow-Derived Mesenchymal Stromal Cells in a Rat Model of Spinal Cord Injury. Acta Neurochir (Wien) 155, 1943–1950. 10.1007/s00701-013-1799-5 23821338

[B70] Tao G. Zhang N. Shang Z. Zhang Y. Zhang T. Zhang J. (2019). Interpretation on Examples of SYRCLE'tool for Interviewing Risk of Bias in Animal Experimentation. Chin. J. Evid. Based Cardiovasc. Med. 11, 292–295.

[B71] Tator C. H. (2006). Review of Treatment Trials in Human Spinal Cord Injury: Issues, Difficulties, and Recommendations. Neurosurgery 59, 957–7. 10.1227/01.NEU.0000245591.16087.89 17143232

[B72] Tator C. H. (1995). Update on the Pathophysiology and Pathology of Acute Spinal Cord Injury. Brain Pathol. 5, 407–413. 10.1111/j.1750-3639.1995.tb00619.x 8974623

[B73] Vaquero J. Zurita M. Oya S. Santos M. (2006). Cell Therapy Using Bone Marrow Stromal Cells in Chronic Paraplegic Rats: Systemic or Local Administration. Neurosci. Lett. 398, 129–134. 10.1016/j.neulet.2005.12.072 16423458

[B74] Vawda R. Wilcox J. Fehlings M. (2012). Current Stem Cell Treatments for Spinal Cord Injury. Indian J. Orthop. 46, 10–18. 10.4103/0019-5413.91629 22345801 PMC3270592

[B75] Vismara I. Papa S. Rossi F. Forloni G. Veglianese P. (2017). Current Options for Cell Therapy in Spinal Cord Injury. Trends Mol. Med. 23, 831–849. 10.1016/j.molmed.2017.07.005 28811172

[B76] Volarevic V. B. S. Markovic M. A. Gazdic N. N. Volarevic L. V. Jovicic M. M. Arsenijevic N. (2018). Ethical and Safety Issues of Stem Cell-Based Therapy. Int. J. Med. Scis 15, 36–45. 10.7150/ijms.21666 PMC5765738 29333086

[B77] Wu Y. X. Kang D. Y. Hong Q. Wang J. L. (2011). External Validity and its Evaluation Used in Clinical Trials. Zhonghua Liu Xing Bing Xue Za Zhi 32, 514–518. 21569739

[B78] Yousefifard M. Nasseri Maleki S. Askarian-Amiri S. Vaccaro A. R. Chapman J. R. Fehlings M. G. (2019). A Combination of Mesenchymal Stem Cells and Scaffolds Promotes Motor Functional Recovery in Spinal Cord Injury: a Systematic Review and Meta-Analysis. J. Neurosurg. Spine 32, 269–284. 10.3171/2019.8.SPINE19201 31675724

[B79] Yousefifard M. Rahimi-Movaghar V. Nasirinezhad F. Baikpour M. Safari S. Saadat S. (2016). Neural Stem/progenitor Cell Transplantation for Spinal Cord Injury Treatment; A Systematic Review and Meta-Analysis. Neuroscience 322, 377–397. 10.1016/j.neuroscience.2016.02.034 26917272

[B80] Zeng X. Xiong Q. Shen K. (2013). Meta-analysis Series Thirteen: Evaluation of Blind Method. Chin. J. Evid. Based Cardiovasc. Med. 5, 331–333.

[B81] Zholudeva L. V. Lane M. A. (2019). Transplanting Cells for Spinal Cord Repair: Who, what, when, where and Why. Cel Transpl. 28, 388–399. 10.1177/0963689718824097 PMC6628562 30654638

