# Supplementary material for: Spinal Cord Injury: A Systematic Review and Network Meta-Analysis of Therapeutic Strategies Based on 15 Types of Stem Cells in Animal Models
Source: Front Pharmacol. 2022 Mar 14;13:819861. doi: 10.3389/fphar.2022.819861 (PMC8964098; doi:10.3389/fphar.2022.819861)
Supplement: Supplementary file 1 [file DataSheet1.docx]

**Spinal cord injury: a systematic review and network meta-analysis of therapeutic strategies based on 15 types of stem cells in animal models**

**Catalogue**

**Table 1: Chinese and English search strategies**

**Table 2: Basic information of included studies**

**Table 3:** **Meta - analysis results of direct comparison 1 week after stem cell therapy**

**Table 4:** **Meta-analysis results of direct comparison 3 week after stem cell therapy**

**Table 5:** **Meta-analysis results of direct comparison 5 week after stem cell therapy**

**Table 6:** **Meta-analysis results of direct comparison 8 week after stem cell therapy**

**Table 7:** **Ranking plot of therapeutic potential in the first week after stem cell therapy**

**Table 8:** **Ranking plot of therapeutic potential in the third week after stem cell therapy**

**Table 9:** **Ranking plot of therapeutic potential in the fifth week after stem cell therapy**

**Table 10:** **Ranking plot of therapeutic potential in the eighth week after stem cell thera**

**Table 1: Chinese and English search strategies**

| Comments:  In order for non-Chinese readers to understand the Chinese search strategy of this article, we translated the Chinese search terms in the search formula.  **PubMed 2021.09.14**  #1: "Stem Cells"[Mesh] OR "Stem Cell Research"[Mesh] 235,173  #2: "stem cell"[Title/Abstract] OR "stem cells"[Title/Abstract] 296,835  #3: #1 OR #2 386,274  #4: "Spinal Cord Injuries"[MeSH Terms] OR "Spinal Injuries"[MeSH Terms] 73,029  #5: "spinal cord injury"[Title/Abstract] OR "spinal injury"[Title/Abstract] OR "spinal cord trauma"[Title/Abstract] OR "spinal cord transection"[Title/Abstract] OR "spinal cord laceration"[Title/Abstract] OR "post traumatic myelopathy"[Title/Abstract] OR "spinal cord contusion"[Title/Abstract] 41,813  #6: #4 OR #5 85,995  #7: #3 AND #6 2,867  **WOS (Source of literature:Web of Science Core Collection 8,229;MEDLINE® 7,673;BIOSIS Previews 6,814;KCI-Korean Journal Database114;SciELO Citation Index 34;Russian Science Citation Index 20）2021.09.14**  (TS= (Spinal cord injury OR Spinal injury OR Spinal Cord Trauma OR Spinal Cord Transection OR Spinal Cord Laceration OR Post-Traumatic Myelopathy OR Spinal Cord Contusion)) AND TS= (stem cell OR stem cells) 8976  **EMBASE 2021.09.14**  #1: stem cell. ab,kw,ti. 281680  #2: stem cell/ 139583  #3: spinal cord injury/ 59998  #4: spine injury/ 10674  #5: spinal cord transsection/ 3177  #6: (Spinal cord injury or Spinal injury or Spinal Cord Trauma or Spinal Cord Transection or Spinal Cord Laceration or Post-Traumatic Myelopathy or Spinal Cord Contusion).au,kw,ti. 39525  #7: 1 or 2 361729  #8: 3 or 4 or 5 or 6 77853  #9: 7 and 8 2436  **CNKI/高级检索:** **2021.09.14; CNKI / Advanced Search: 2021.09.14 (文献来源:SCI来源期刊 EI来源期刊 核心期刊 CSSCI CSCD)**  主题:脊髓损伤 AND (干细胞 OR 万能细胞 OR 祖细胞) (1010)  Subject: spinal cord injury AND (stem cell OR stem cells) (1010)  **万方/高级检索: 2021.09.14; Wanfang database/ Advanced Search: 2021.09.14**  主题:脊髓损伤 AND (干细胞 OR 万能细胞 OR 祖细胞) (1752)  Subject: spinal cord injury AND (stem cell OR stem cells) (1752)  **VIP/高级检索: 2021.09.14; VIP database/ Advanced Search: 2021.09.14**  题名或关键词: 脊髓损伤 AND (干细胞 OR 万能细胞 OR 祖细胞) (590)  Title or keyword: spinal cord injury AND (stem cell OR stem cells) (590)  **CBM/高级检索:** 2021.09.14; CBM / Advanced Search: 2021.09.14  #1:"脊髓损伤"[不加权:扩展] 60530  #2:"脊髓损伤"[常用字段:智能] 78047  #3:#2 OR #3 78047  #4:"干细胞"[不加权:扩展] 354238  #5:"干细胞"[常用字段:智能] OR "万能细胞"[常用字段:智能] OR "祖细胞"[常用字段:智能] 671774  #6:#4 OR #5 671774  #7:#3 AND #6 1579  #1:" Spinal cord injury "[unweighted, extended] 60530  #2:" Spinal cord injury "[ common field: smart] 78047  #3:#2 OR #3 78047  #4:"Stem cell"[unweighted, extended] 354238  #5:" Stem cell "[ common field: smart] OR " stem cells"[ common field: smart] OR " progenitor cell "[ common field: smart] 671774  #6:#4 OR #5 671774  #7:#3 AND #6 1579 |
| --- |

**Table 2: Basic information of included studies**

| **Number** | **Author(year)** | **Country** | **Type of study** | **Baseline characteristics** | | | | **Species size** | **Model** | | | **Characteristics of stem cells** | | **Administration** | **Injection time (after SCI)** | **Interventions** | |
| --- | --- | --- | --- | --- | --- | --- | --- | --- | --- | --- | --- | --- | --- | --- | --- | --- | --- |
|  |  |  |  | **Species** | **Breed** | **Body weight** | **Age** | **Experience/Control** | **Modeling** | **Injury pathway** | **Type of model** | **Species** | **Sources** |  |  | **Stem cells** | **Negative control** |
| 1 | BASAK 2021 | Turkey | RCT | Wistar-Hnorver rats | Female | 225-275g | Adult | 8/8 | Complete spinal cord transection at the T8 lamina level. | Transection | Acute | BMSCs | Wistar Hannover female rats | Spinal cord | Immediately | 0.5×10^6^ | Blank |
| 2 | Zou 2020 | China | RCT | SD rats | Female | / | 6-8weeks | 25/25/25 | Complete spinal cord transection at the T9 lamina level. | Transection | Acute | UCMSCs; NSCs | The aborted fetus | Spinal cord | Immediately | 2×10^6^ | Blank |
| 3 | SAPORTA 2003 | America | RCT | SD rats | Male | / | Adult | 18/18 | Clamp the dorsal and ventral spinal cord with aneurysm clip (55 g) at T8 / 9 level (1 min ). | Extrusion | Subacute | UCMSCs | Human cord blood | Caudal vein | 5 days after operation | 1×10^6^ | Blank |
| 4 | Zhang 2013 | China | RCT | SD rats | Male | 200-230g | 10-12weeks | 6/6 | Extrusion 5 min at T8 level at about 20 g weight and 0.5 mm / min vertical speed. | Extrusion | Subacute | BMSCs | Bone marrow of rat femur and tibia | Spinal cord | 5 days after operation | 4×10^5^ | Blank |
| 5 | Na 2020 | China | RCT | SD rats | Male | 220-260g | / | 10/10 | SCI caused by free fall from a height of 10.00 mm with 10g impact bar at T10 level. | Contusion | Acute | UCMSCs | Human cord | Spinal cord | Immediately | 1×10^6^ | Blank |
| 6 | Ohta 2004 | Japan | RCT | SD rats | / | 70-90g | 4weeks | 18/18 | Drop 10g metal rod from 12.5 mm (mild contusion) or 25.0 mm (severe contusion) to spinal cord exposure at T8-T9 level. | Contusion | Acute | BMSCs | Bone marrow of SD rats | Spinal cord | Immediately | 5×10^6^ | Blank |
| 7 | Watanabe 2004 | Japan | RCT | SD rats | Female | 250-300g | Adult | 18/10 | Drop 10 grams of weight from 25 mm to dura at T10 level. | Contusion | Subacute | NSCs | Embryos of the SD rats | Spinal cord | 9 days after operation | 1×10^8^ | Blank |
| 8 | Cízková 2006 | Slovakia | RCT | Wistar rats | Male | 300-320g | Adult | 15/15 | Balloon compression technique caused SCI. | Contusion | Subacute | NSCs | Human iliac-derived bone marrow MSCs | Femoral vein | 7 days after operation | 1×10^6^ | DMEM |
| 9 | Himes 2006 | America | RCT | SD rats | Female | 225-250g | Adult | 7/8 | The animal was immobilized in a Mascis impactor and fell from a predetermined height with a 10g stick. | Contusion | Subacute | BMSCs | Adult bone marrow | Spinal cord | 7 days after operation | 5×10^5^ | Vehicle |
| 10 | Urdzíková 2006 | Czech Republic | RCT | Wistar rats | Male | 300-330g | 4weeks | 15/15 | SCI induced by balloon at T8-9 level. | Extrusion | Subacute | BMSCs | Femur and tibia of the Wistar rats | Femoral vein | 7 days after operation | 2×10^6^ | Normal saline |
| 11 | NISHIO 2006 | Japan | RCT | Wistar rats | Male | 205-245g | 8-10weeks | 8/11 | 10 g weight downed from 25 mm to spinal cord at T9 level. | Contusion | Subacute | HSCs | The umbilical cord of the fetus | Spinal cord | 7 days after operation | / | Vehicle |
| 12 | Parr 2007 | Canada | RCT | SD rats | Female | 250-300g | / | 8/9 | Injured 1 min with 35 g force clip at T8-9 level. | Extrusion | Acute | NSCs | The ependymal zone of adult male Wistar rats | Spinal cord | Immediately | / | Blank |
| 13 | Cho 2008 | Korea | RCT | SD rats | Male | 300-350g | Adult | 13/20 | Use the NYU weight-drop device at the T9 level to drop a 10 g impact bar from a height of 25 mm onto the exposed surface of the dorsal spinal cord. | Contusion | Subacute | UCMSCs | The umbilical cord of the fetus | Spinal cord | 7 days after operation | 2×10^5^ | PBS |
| 14 | Deng 2008 | China | RCT | SD rats | Female | 240-270g | / | 15/15/15 | Drop 10 g impact rod from 50 mm at T9-10 level. | Contusion | Acute | BMSCs+OECs | Human iliac bone marrow, aborted or dead fetal outer olfactory bulb | Spinal cord | 30min after operation | 2.5×10^5^ | DMEM |
| 15 | Li 2008 | China | RCT | Wistar rats | Male | 200±20g | 16weeks | 5/5 | Spinal cord injury caused by compression of 30 g arterial clips at T10 for 30 seconds. | Extrusion | Acute | BMSCs | Bone marrow of SD rats | Spinal cord | Immediately | 1×10^5^ | PBS |
| 16 | SHETH 2008 | America | RCT | SD rats | Female | 160-180g | Adult | 24/14 | At T9 level, the NYU weight-drop device dropped the 10g impact bar from 12.5mm height onto the exposed surface of the spinal dorsal. | Contusion | Subacute | BMSCs | Human bone marrow | Spinal cord | 7 days after operation | 6×10^5^ | PBS |
| 17 | Yang 2008 | China | Control | SD rats | Female | 250-300g | Adult | 13/8 | Complete spinal cord transection with a scalpel after laminectomy at thoracic segments 7-9. | Transection | Acute | UCMSCs | Wharton’s jelly of the umbilical cord of human | Spinal cord | Immediately | 5×10^5^ | Blank |
| 18 | Chiba 2008 | Japan | Control | SD rats | Female | 200-250g | Adult | 7/7 | SCI caused by impact device (2 m / s) at T10 level. | Contusion | Acute | BMSCs | Femur bone marrow of SD rats | Spinal cord | Immediately | 7×10^4^ | Vehicle |
| 19 | Cho 2009 | Korea | RCT | SD rats | / | 300-350g | Adult | 15/13 | NYU impactor to create a free drop contusion at the T9 level. | Contusion | Subacute | BMSCs | Femur bone marrow of SD rats | Spinal cord | 7 days after operation | 2.5×10^5^ | PBS |
| 20 | Liang 2009 | China | RCT | Wistar rats | Female | 180-200g | 5-7weeks | 12/6 | After laminectomy was performed at the T9 level, the dura was opened and the spinal cord was transected using iridectomy scissors. | Transection | Acute | BMSCs | Femur and tibia of the Wistar rats | Spinal cord | Immediately | 5×10^5^ | Blank |
| 21 | Guo 2007 | China | RCT | SD rats | Female | 200-220g | Adult | 10/10 | A laminectomy was carried out to expose the T9and T10 spinal segment. Spinal cord (2 mm) was transected and cut away between T9 and T10segment. | Transection | Acute | NSCs | Brain tissue of neonatal SD rats | Spinal cord | Immediately | 4×10^6^ | Blank |
| 22 | Sasaki 2009 | America | Control | SD rats | Female | 150-179g | Adult | 16/6 | T9 laminectomy was performed, and the dorsal funiculus was transected using an ophthalmic microscalpel. | Transection | Acute | BMSCs | Human iliac bone marrow | Spinal cord | Immediately | 1.2×10^5^ | Blank |
| 23 | Shen 2009 | China | RCT | SD rats | Female | 230-250g | Adult | 12/12 | After laminectomy, the impact rod weighted 10 g was dropped from a height of 25 mm above Th9 spinal level. | Contusion | Subacute | BMSCs | Femur and tibial bone marrow of SD rats | Spinal cord | 7 days after operation | 1×10^5^ | PBS |
| 24 | Alexanian 2010 | America | RCT | SD rats | Female | 200-250g | Adult | 10/10 | NYU Impactor was used to produce a consistent, uniformly moderate injury (10-g drop from a height of 25 mm directly onto the dura at level T8). | Contusion | Subacute | BMSCs | Femur bone marrow of SD rats | Spinal cord | 7 days after operation | 1×10^5^ | PBS |
| 25 | AMEMORI 2010 | Czech Republic | RCT | Wistar rats | Male | 270-300g | Adult | 23/23 | Balloon compression was used to create an SCI. | Extrusion | Subacute | BMSCs | The ff mucosa and tibia, femur in Wistar rats | Spinal cord | 7 days after operation | 1×105 | Normal saline |
| 26 | Gu 2012 | China | Control | SD rats | / | / | Adult | 25/25 | Rats were subjected to complete spinal cord transection at the eighth thoracic. | Transection | Acute | NSCs | The hippocampal tissue of (GFP) transgenic embryonic mouse | Spinal cord | Immediately | 3×10^5^ | Blank |
| 27 | Fang 2010 | China | Control | SD rats | Female | 250±20g | Adult | 6/3 | A laminectomy was performed at T9-T10, and the dorsal surface of the spinal cord was compressed by dropping a 10-gm rod from a height of 50 mm. | Contusion | Subacute | BMSCs | Bone marrow of SD rats | Spinal cord | 7 days after operation | 2×10^5^ | Vehicle |
| 28 | Ide 2010 | Japan | RCT | SD rats | Female | / | 4-6weeks | 48/42 | The spinal cord covered by dura mater was crush-injured by spontaneously dropping a 10 g metal rod from a height of 7.5 cm using an NYU impactor at Th8-9. | Contusion | Subacute | BMSCs | The femurs and tibias of GFP-transgenic SD rats | Spinal cord | 14 days after operation | 5×10^5^ | PBS |
| 29 | Pourheydor 2012 | Iran | RCT | Wistar rats | Female | 250-300g | Adult | 8/8 | A mental rod 10g in weight and 2mm in diameter was dropped from a height of 12.5mm onto the exposed spinal cord at the T8 level. | Contusion | Subacute | BMSCs | Bone marrow of SD rats | Spinal cord | 7 days after operation | 3×10^5^ | Blank |
| 30 | Lebedev 2010 | Russia | RCT | albino rats | Male | 300-350g | / | 30/23 | Contusion of the spinal cord was modeled with a metal rod (2 mm in diameter, 10 mg) fixed in a stereotaxis micromanipulator and falling vertically from a height of 12.5 mm at T8-T10. | Contusion | Acute | NSCs | Human sniffing mucous membrane | Spinal cord | Immediately | 7.5×10^5^ | PBS |
| 31 | Osaka 2010 | Japan | RCT | SD rats | / | 250-300g | Adult | 20/20 | Contusion injury was induced using a 10 g weight dropped at 55 mm onto Th9–10 spinal cord exposed by laminectomy by using the NYU weight drop impactor. | Contusion | Subacute | BMSCs | Bone marrow of the femur of the SD rats | Vein | 7 days after operation | 1×10^6^ | Normal saline |
| 32 | Pedram 2010 | Iran | RCT | Wistar rats | Male | 300-350g | 8-12weeks | 6/5 | The balloon was rapidly inflated with 20ml volume of saline for 5 min at T8-T9 level. | Extrusion | Subacute | BMSCs | The femur bone marrow of the same rat | Spinal cord | 7 days after operation | 1×10^6^ | Blank |
| 33 | Wang 2010 | China | Control | SD rats | Male | 200-250g | / | 6/6 | Spinal cord injury at T9 level. | Contusion | Subacute | NSCs; OECs | Brain cortex and olfactory bulb of the adult rats | Spinal cord | 7 days after operation | 1×10^5^ | DMEM |
| 34 | He 2013 | China | RCT | SD rats | / | / | Adult | 25/25 | Complete spinal cord transection at the thoracic spinal cord (T10). | Transection | Acute | NSCs | Hippocampal tissue of green fluorescent protein (GFP) transgenic embryonic mouse | Spinal cord | Immediately | 3×10^5^ | Blank |
| 35 | Cizkova 2010 | Czechoslovakia | RCT | Wistar rats | Male | 300-320g | Adult | 8/8/8 | SCI induced by balloon compression technique. | Extrusion | Acute | BMSCs | Femur and tibial bone marrow of Wistar rats | Spinal cord | Immediately | 5×105;1.5×106 | Normal saline |
| 36 | Ding 2011 | China | Control | SD rats | Female | 230-250g | Adult | 18/13 | Transverse spinal cord at T10 level. | Transection | Acute | BMSCs | Femur and tibial bone marrow of SD rats | Spinal cord | Immediately | 5×10^5^ | Blank |
| 37 | Li 2011 | China | RCT | SD rats | Female | 200-250g | Adult | 5/5 | The spinal cord at T9was then cut transversely and a 1–2 mm tissue block removed with an iris razor blade to ensure complete transection of the spinal cord. | Transection | Acute | NSCs | The hippocampi of Green fluorescent protein (GFP) transgenic pregnant mice | Spinal cord | Immediately | 6×10^5^ | Blank |
| 38 | Liu 2011 | China | RCT | SD rats | Male | 200-220g | Adult | 24/24 | Under sterile conditions, rat spinal cords were injured at T10 through an 8 g×2.5 cm force, which resulted in complete paraplegia. | Extrusion | Acute | BMSCs | / | Spinal cord | 10min after operation | 1×10^6^ | DMEM |
| 39 | Seo 2011 | Korea | RCT | SD rats | Male | 300-350g | / | 12/13 | SCI was induced by dropping a 10-g impact rod from a 25-mm height onto the exposed dorsal surface of the spinal cord of the rats using the NYU impactor (New York University, NY) at T9 level. | Contusion | Acute | UCMSCs | Human cord blood | Caudal vein | Immediately | 1×10^6^ | PBS |
| 40 | Wang 2011 | China | Control | SD rats | Female | 220-250g | Adult | 10/10 | Following laminectomy at T9-10, the spinal cord was transected at T10 with a sharp tip surgical scalpel. | Transection | Acute | NSCs | Hippocampal tissue of SD rats | Spinal cord | Immediately | 1×10^6^ | Culture medium |
| 41 | Wu 2011 | China | Control | SD rats | / | / | / | 15/15 | Contused incomplete SCI was induced by dropping a 10-g weight rod from a 6 cm height onto the exposed dorsal surface of the spinal cord using an NYU impactor at T10 level. | Contusion | Subacute | BMSCs | SD rats | Spinal cord | 7 days after operation | 5×10^5^ | PBS |
| 42 | Yan 2011 | China | RCT | SD rats | Female | 220-250g | / | 5/5 | A laminectomy was carried out at the T8–T10 level to expose the T10 spinal segment. The dura was cut and the T10 spinal segment was transected completely. | Transection | Acute | BMSCs | Femur of SD rats | Spinal cord | Immediately | 5×10^5^ | Blank |
| 43 | Cheng 2012 | America | RCT | Long-Evans hooded rats | Female | 200-350g | Adult | 6/6 | Moderate spinal cord contusion was induced by the Multicenter Animal Spinal Cord Injury Study Impactor with 10-g weight dropped from a height of 25 mm. | Contusion | Acute | NSCs | Human fetal brain tissue | Spinal cord | Immediately | 5×10^5^ | Culture medium |
| 44 | Kang 2012a | Korea | RCT | SD rats | Male | 250-300g | Adult | 12/12 | All the spinal contusions were induced by a 25 g-cm contusion using the MASCIS (Multicenter Animal Spinal Cord Injury Study) impactor (a rod weighing 10 g and dropped from a height of 2.5 cm). | Contusion | Acute | BMSCs | Femur of SD rats | Caudal vein; Spinal cord | 1 days after operation | 1×10^6^ | PBS |
| 45 | karaoz 2011 | Turkey | Control | Wistar rats | / | 200-300g | 8weeks | 3/3 | For SCI groups, a severe T10–T11 contusive injury was introduced by dropping the impounder rod (1 g) from a height of 50 mm. | Contusion | Acute | BMSCs | Rat femur and tibial bone marrow | Spinal cord | Immediately | 3×10^5^ | PBS |
| 46 | Taghipour 2012 | Iran | RCT | Wistar rats | Male | 250-300g | / | 10/10 | The contusion injury was carried out by dropping a 10 g weight rod from a 25-mm height onto the dorsal surface of the spinal cord (NYU impactor). | Contusion | Subacute | DPSCs | People's deciduous teeth | Spinal cord | 7 days after operation | 5×10^5^ | PBS |
| 47 | Liu 2012 | China | RCT | SD rats | Male | / | Adult | 30/30 | Contused incomplete SCI was induced by dropping a 10-g weight rod from a 6-cm height onto the exposed dorsal surface of the spinal cord using an NYU impactor. | Contusion | Subacute | BMSCs | Bone marrow of the femur of the SD rat | Spinal cord | 7 days after operation | 5×10^5^ | PBS |
| 48 | Nakajima 2012 | Japan | RCT | SD rats | Male | 271±29.1g | Adult | 5/5 | At the T9–T10 vertebral level, the dorsal surface of the spinal cord was compressed extramurally using the Infinite Horizons Impactor, with an impact force of 200 kilodynes (kdyn). | Contusion | Acute | BMSCs | Human bone marrow | Spinal cord | 3 days after operation | 1×10^6^ | Culture medium |
| 49 | Oh 2012 | Korea | RCT | SD rats | Male | 250-300g | Adult | 10/10 | The spinal cord was injured by clip compression at the T9 level for 10min. | Extrusion | Acute | ADMSCs | Human abdominal fat | Spinal cord | Immediately | 3×10^5^ | PBS |
| 50 | Park 2012 | Korea | RCT | SD rats | Male | 270-300g | Adult | 26/12 | The impact rod of the NYU impactor was centered above T9 and dropped from a height of 25mm to induce an incomplete partial SCI. | Contusion | Subacute | BMSCs | Human cord blood mesenchymal stem cells | Spinal cord | 7 days after operation | 3×10^5^ | PBS |
| 51 | ODA 2013 | Japan | RCT | SD rats | / | 250-300g | 8weeks | 8/8 | / | Transection | Acute | BMSCs | Bone marrow of the femur of the SD rat | Spinal cord | Immediately | 1.2×10^6^ | DMEM |
| 52 | Quertainmont 2012 | Belgium | RCT | Wistar rats | Female | 250g | Adult | 20/10 | A 2-French Fogarty arterial embolectomy catheter was inserted in the epidural space at the T10 level with a distilled water volume of 15ml and left in place during 5 minutes. | Extrusion | Subacute | BMSCs | Bone marrow of tibia and femur in Wistar rats | Spinal cord | 7 days after operation | 1×10^6^ | Blank |
| 53 | Sakai 2012 | Japan | Control | SD rats | Female | / | Adult | 10/10 | The SC was completely transected using a surgical blade. | Transection | Acute | DPSCs; BMSCs | Human deciduous teeth | Spinal cord | Immediately | 1×10^6^ | Blank |
| 54 | Xu 2012 | China | Control | Wistar rats | / | 200-250g | Adult | 30/30 | Contusion injury was induced using a 20 g weight to drop from 10 cm high onto the surface of Th9-10 spinal cord exposed by laminectomy (NYU weight drop impactor). | Contusion | Subacute | NSCs | Wistar rats | Caudal vein | 7 days after operation | 1×10^6^ | Normal saline |
| 55 | Yazdani 2012 | Iran | RCT | Wistar rats | Female | 300-350g | Adult | 7/7 | The center of the balloon rested at T8–T9 level of the spinal cord. The balloon was then rapidly inflated with a defined volume of saline (15 μl) for 5 min. | Extrusion | Subacute | OECs; BMSCs | Bone marrow of tibia and femur in Wistar rats | Spinal cord | 7 days after operation | 1×10^6^ | Culture medium |
| 56 | Zhou 2012 | China | RCT | SD rats | Female | 200-250g | / | 8/8 | The dura matter covered spinal cord was crush-injured by spontaneously dropping a 10 g metal rod from a height of 7.5 cm using an NYU impactor. | Extrusion | Acute | UCMSCs | Human cord | Spinal cord | Immediately | 2×10^5^ | PBS |
| 57 | Li 2014a | China | Control | Wistar rats | / | 250~300g | 8weeks | 21/21 | The spinal cord was transected at the level of T9–10, leaving a 2-mm gap between the proximal and distal ends of the resected cord. | Transection | Acute | BMSCs | The tibia of Wistar rats | Caudal vein | 6h after operation | 5×10^6^ | Culture medium |
| 58 | Liu 2013a | China | RCT | SD rats | / | / | / | 15/15 | A weight of 10 g was dropped from a height of 50 mm onto the exposed spinal cord and the impounder was left for 20 sec before withdrawal. | Contusion | Acute | BMSCs | Tibia and femur of SD rats | Spinal cord | 1 days after operation | 5×10^4^ | PBS |
| 59 | Kim 2013 | Korea | RCT | SD rats | Male | 290-340g | Adult | 12/12 | Contusion on the spinal cord was induced by a 25 g-cm contusion using the MASCIS impactor (a rod weighing 10 g and dropped from a height of 2.5 cm) at the T9 level. | Contusion | Chronicity | BMSCs | Femur of SD rats | Caudal vein; Spinal cord | 6 weeks after operation | 1×10^6^ | Blank |
| 60 | Shin 2013 | Korea | RCT | SD rats | Male | 300-350g | Adult | 24/24 | A rat model of spinal cord injury was produced by dropping a 10-g weight, 2 mm in diameter, onto the exposed spinal cords of animals from a height of 25 mm. | Contusion | Subacute | BMSCs | Human iliac bone | Caudal vein; Spinal cord | 7 days after operation | Vein:2×10^6^; Spinal cord:3×10^5^ | Normal saline |
| 61 | Zhou 2013 | China | Control | SD rats | Female | 200-250g | 8weeks | 5/5 | Dorsal columns were cut with a pair of micro scissors to the depth of the central canal. | Transection | Acute | ADMSCs; BMSCs | Adult bone marrow and subcutaneous fat | Spinal cord | Immediately | 2×10^5^ | PBS |
| 62 | Lin 2013 | China | RCT | SD rats | / | 200-250g | / | 24/24 | Balloon angioplasty catheters filled with normal saline were inserted into the epidural spaces from the T10 level to the T9 level for 5 minutes. | Extrusion | Acute | BMSCs | Femur of SD rats | Spinal cord | 1 days after operation | 5×10^6^ | Normal saline |
| 63 | Hodgetts 2013 | Australia | RCT | CBH-rnu/Arc (Athymic Nude) rats | Female | 120-150g | Adult | 8/8 | Using an NYU impactor, a moderate contusion injury was induced by dropping a 10-g weight from 12.5 mm above the exposed SC. | Contusion | Subacute | BMSCs | Human iliac bone | Spinal cord | 7 days after operation | 5×10^5^ | Blank |
| 64 | Ning 2013 | China | RCT | Wistar rats | Female | 220±20g | Adult | 40/40 | Placing the rat on the MASCIS Impactor machine, its exposed spinal cord was hit by the 10 g (weight) by 50 mm (height) weight drop. | Contusion | Acute; Subacute | UCMSCs | Human umbilical cord blood | Spinal cord | 1 and 6 days after operation | 7.5×10^5^ | DMEM |
| 65 | CUI 2014 | China | RCT | Wistar rats | Female | 250-280g | Adult | 16/15 | The exposed spinal cord at the T9 level was vulnerated with a 10 g weight dropped from a height of 2.5 cm (vulnerating energy, 25 g/cm). | Contusion | Acute | UCMSCs | Human umbilical cord blood | Spinal cord | Immediately | 5×10^4^ | Normal saline |
| 66 | Li 2014b | China | RCT | SD rats | Male | 250±25g | Adult | 30/30 | Modified Allen method. | Contusion | Acute | NSCs | SD rats | Caudal vein | 1 days after operation | 5×10^6^ | PBS |
| 67 | Judas 2014 | Brazil | RCT | Wistar rats | Male | 350-400g | / | 10/10 | Spinal cord ischemia was induced by intraluminal balloon occlusion of the descending thoracic aorta jointly with left subclavian artery. | Ischemia | Acute | UCMSCs | Human umbilical cord blood | Spinal cord | 30min after operation | 1×10^4^ | PBS |
| 68 | Ryabov 2014 | Russia | Control | SD rats | Male | 250-300g | / | 6/11 | Contusion was inflicted by a metal rod (2 mm in diameter, 10 g) vertically dropped from the height of 25 mm. | Contusion | Acute | UCMSCs | Human umbilical cord blood | Caudal vein | 1 days after operation | 1×10^7^ | Normal saline |
| 69 | Liu 2013b | China | RCT | SD rats | Female | 250-300g | Adult | 22/10 | The rod of the impactor (10 g) was centered above T10 and dropped from a height of 25 mm to induce a consistent partial and incomplete spinal cord injury. | Contusion | Acute; Subacute | NSCs | Embryonic brain tissue of SD rats | Subarachnoid space | 1h and 7 days after operation | 4×10^6^ | Blank |
| 70 | Mitsuhara 2013 | Japan | RCT | Fischer/F344 rats | Female | 150-200g | Adult | 11/7 | A cylindrical brass weight (10 g) was dropped down a stationary rod onto an impactor rod that rested on surface of the T12 dorsal dura mater. | Contusion | Acute | BMSCs | Bone marrow of femur and tibia of Fischer / F344 rats | Caudal vein | Immediately | 3×10^5^ | PBS |
| 71 | Ormond 2014 | America | RCT | SD rats | Female | 200-250g | Adult | 7/6 | A guided 10 g rod was dropped 12.5 or 25 mm onto the exposed dura mater, representing moderate or severe SCI, respectively. | Contusion | Subacute | NSCs | Subventricular zone of SD rats | Spinal cord | 7 days after operation | 1×10^6^ | DMSO |
| 72 | Hong 2014 | Korea | RCT | SD rats | Female | 230-250g | Adult | 28/23 | A 10-g rod was dropped from 25-mm height onto the exposed T9 spinal cord and allowed to rest for 5 s. | Contusion | Subacute | NSCs | The brain tissue of 16.5 OG2/ROSA26 heterozygous female mice | Spinal cord | 9 days after operation | 1×10^6^ | Vehicle |
| 73 | Chen 2014 | China | RCT | SD rats | / | 180-200g | Adult | 6/6 | The vertebral bodies at T9 and T11 were grasped with 2 adjustable forceps to immobilize the spine. The impact force of the impactor was set at 200 kDynes. | Contusion | Subacute | BMSCs | Tibia and femur of SD rats | Caudal vein | 7 days after operation | 1×10^6^ | DMEM |
| 74 | Aizawa-Kohama 2013 | Japan | RCT | Wistar rats | Female | 200±20g | 10weeks | 10/14 | Possible remaining adhesions were cut with a scalpel, and the rostral and caudal stumps were carefully lifted to verify complete transection. | Transection | Subacute | NSCs | Wistar rats | Spinal cord | 9 days after operation | 3×10^5^ | Vehicle |
| 75 | Li 2014c | China | RCT | Wistar rats | Half male and half female | 240±10g | 10weeks | 6/6 | Using the NYU impactor method, the spinal cord injury was produced by dropping a 10-g weight from a height of 25 mm. | Transection | Acute | PDMSCs | Placenta tissue of healthy parturient | Spinal cord | Immediately | 1×10^6^ | Normal saline |
| 76 | Hofstetter 2005 | Sweden | RCT | SD rats | Female | 250g | Adult | 20/28 | In rats sedated by halothane anesthesia, the spinal cord was exposed by a laminectomy of T8–9 and was subjected to impact by a weight dropped from a height of 12.5 mm. | Contusion | Subacute | NSCs | / | Spinal cord | 7 days after operation | 1×10^5^ | Vehicle |
| 77 | Hosseini 2016 | Iran | RCT | Wistar rats | Male | 190-220g | Adult | 7/5 | The animals were subjected to an impact of 10 g weight (stainless steel rod, 3 mm diameter tip) dropped vertically in the center of the exposed spinal cord from the height of 25 mm. | Contusion | Acute | UCMSCs | Umbilical cord of healthy pregnant women | Spinal cord | 1 days after operation | 3×10^5^ | Normal saline |
| 78 | Chen 2015 | China | RCT | Wistar rats | / | / | / | 12/12 | A 10-g weight was allowed to drop from 12.5 mm onto the T-10 segment resulting in a moderate SCI. | Contusion | Acute | BMSCs | Tibia and femur of SD rats | Spinal cord | Immediately | 3×10^5^ | Blank |
| 79 | Song 2015 | China | RCT | SD rats | Female | 250-300g | 10weeks | 20/20 | The T9-T11 vertebral plates were removed to expose the spinal cord, and the model was established with a modified Allen method. | Contusion | Acute | NSCs | Brain tissue of SD rat fetus | Spinal cord | 6h after operation | 1×10^5^ | PBS |
| 80 | Sarveazad 2014 | Iran | RCT | Wistar rats | Male | 250-350g | Adult | 6/6 | Metal cylinder (weighing 10 g and 2 mm in diameter) was released on the exposed spinal cord from distance of 12.5 cm. | Contusion | Subacute | ADMSCs | Human abdominal adipose tissue | Spinal cord | 7 days after operation | 1×10^6^ | Blank |
| 81 | Geng 2015 | China | RCT | SD rats | Male | 220-250g | 8weeks | 28/28 | / | Not reported | Acute | BMSCs | Bone marrow of tibia and femur in SD rats | Spinal cord | 6h after operation | 1×10^5^ | Normal saline |
| 82 | Wang 2015a | China | RCT | SD rats | Female | 200-250g | / | 15/15 | Using rat forceps, T8 and T9 spinous processes and lamina were removed, exposing the dura mater. The right side of the spinal cord was then cut. | Transection | Acute | BMSCs | Bone marrow of tibia and femur in SD rats | Caudal vein | Immediately | 1×107 | Blank |
| 83 | Zhang 2015a | China | RCT | SD rats | Male | 200±10g | 8weeks | 20/20 | A 10-g force was fell from the 1.25 cm height, obtaining 10 × 1.25 g/cm falling impact. | Contusion | Acute | NSCs | Brain tissue of Wistar rats | Spinal cord | 3 days after operation | 1×10^5^ | Blank |
| 84 | Wang 2016 | China | RCT | SD rats | Female | 250-350g | Adult | 10/6 | / | Not reported | Subacute | AECs | Healthy term placenta of puerperal | Spinal cord | 7 days after operation | 1×10^6^ | Culture medium |
| 85 | Nicola 2016 | Brazil | RCT | Wistar rats | Male | 200-250g | 8weeks | 11/12 | Laminectomy was performed at thoracic vertebral level 9 (T9), and injury was induced through the drop of a 10 g weight from 25 mm height. | Contusion | Acute | DPSCs | Human deciduous teeth | Spinal cord | 1h after operation | 3×10^5^ | Blank |
| 86 | Urdzíková 2014 | Czech Republic | RCT | Wistar rats | Male | 300±15g | 10weeks | 38/12 | Spinal cord compression was induced by the inflation of the balloon with 15 microliters of saline for 5 min at the T8 spinal level. | Extrusion | Subacute | BMSCs | Bone marrow of healthy adults | Spinal cord | 7 days after operation | / | Normal saline |
| 87 | MORITA 2016 | Japan | RCT | SD rats | Male | 250-350g | Adult | 8/8 | A laminectomy performed at the T9-10 level spinal cord, and a 200-kdyn contusion delivered using the Infinite Horizons impactor. | Contusion | Chronicity | BMSCs | Bone marrow of SD rats | Femoral vein | 10 weeks after operation | 1×10^6^ | DMEM |
| 88 | DePaul 2015 | America | RCT | SD rats | / | 225-249g | / | 19/20 | The animals were situated on the platform, and the 2.5 mm stainless steel impactor tip was positioned over the midpoint of T8 and impacted with 250 kDyne force. | Contusion | Acute | ASCs | Human | Caudal vein | 1 days after operation | 4×10^6^ | Normal saline |
| 89 | Yaghoobi 2016 | Iran | RCT | / | Female | / | Adult | 7/7 | / | Not reported | Acute | UCMSCs | Umbilical cord of healthy pregnant women | Spinal cord | 1 days after operation | 3×10^5^ | Blank |
| 90 | Sandner 2016 | Germany | RCT | Fischer 344rats | Female | / | Adult | 12/13 | At midthoracic level T9 using the Infinite Horizon (IH) Impactor SCI device with an impact force of 200 kilodynes (kdyn). | Contusion | Acute | BMSCs | Bone marrow of femur and tibia of Fischer 344 rats | Spinal cord | 3 days after operation | 1×10^5^ | Blank |
| 91 | Wang 2015b | China | RCT | Wistar rats | Female | 200-250g | 4weeks | 20/20 | In accordance with the modified Allen’s method, 10 g weight freely fell from a height of 2.5 cm and impacted the dura and spinal cord of the rats. | Contusion | Acute | BMSCs | The tibia and femur of Wistar rats | Caudal vein | 6h after operation | 3×10^6^ | Blank |
| 92 | Zhou 2015 | China | RCT | Wistar rats | Female | 200-250g | Adult | 8/8 | According to the modified Allen method, a 10 g weight was dropped from 2.5 cm to directly impact the exposed dura mater and spinal cord tissue. | Contusion | Acute | BMSCs | The tibia and femur of Wistar rats | Caudal vein | 6h after operation | 3×10^6^ | DMEM |
| 93 | Wu 2015 | China | RCT | Wistar rats | Female | 250-350g | Adult | 12/12 | The impact rod of the impactor of 10 g was centered above T10 and dropped from a height of 50 mm to induce a consistent partial SCI. | Contusion | Subacute | BMSCs | Tibia and femur of SD rats | Spinal cord | 7 days after operation | 3×10^5^ | DMEM |
| 94 | Aras 2015 | Turkey | RCT | Wistar rats | Female | 200-220g | 8weeks | 7/7 | T10–T11 injury was induced by clipping (vulnerating energy of 50 g/ cm/60 sec). | Extrusion | Acute | ADMSCs | Peri peritoneal adipose tissue of Wistar rats | Spinal cord | Immediately | 3×10^5^ | Normal saline |
| 95 | Song 2014 | China | RCT | Wistar rats | Male | 160±14g | 8-10weeks | 20/20 | Use the improved aneurysm clamp to remove the T9 segment processes spinous and vertebral lamina, with the calibrated force of 35 g. | Extrusion | Subacute | BMSCs | Femur of Wistar rats | Spinal cord | 7 days after operation | 2.5×10^5^ | Normal saline |
| 96 | Kim 2016 | Korea | RCT | SD rats | Male | 282-322g | Adult | 12/12 | All of the spinal contusions were induced by a 25 g-cm contusion using the MASCIS impactor (a rod weighing 10 g and dropped from a height of 2.5 cm) at the T9 level. | Contusion | Acute | BMSCs | The femur of SD rats | Spinal cord | Immediately | 1×10^6^ | Normal saline |
| 97 | Wang 2014 | China | RCT | SD rats | Female | 180-240g | 8-10weeks | 16/16 | A metal rod with a weight of 25 g and diameter of 2 mm was dropped from a height of 3 cm onto the exposed spinal cord to induce a contusion lesion. | Contusion | Subacute | BMSCs | Tibia and femur of SD rats | Spinal cord | 7 days after operation | 1×10^6^ | Culture medium |
| 98 | Zhang 2015b | China | RCT | SD rats | Male | 180-240g | 8-10weeks | 15/15 | A cylindrical iron rod weighing 25 g was dropped from a height of 30 mm onto the exposed spinal cord to induce a contusion lesion. | Contusion | Subacute | BMSCs | Tibia and femur of SD rats | Subarachnoid space | 7 days after operation | 1×10^6^ | PBS |
| 99 | Ruzicka 2017 | America | RCT | Wistar rats | Male | 3000±15g | 10weeks | 22/25/24/16 | Spinal cord compression was induced by inflation of the balloon with 15 µl of saline for 5 min at the T8 spinal level and then the catheter was emptied. | Extrusion | Subacute | 1.BMSCs ;2. NSCs;3. IPSCs | 1.Human bone marrow; 2. Human embryonic cells, 3. Human | Spinal cord | 7 days after operation | 5×10^5^ | Normal saline |
| 100 | Peng 2017 | China | RCT | SD rats | Male | / | Adult | 18/18 | The T9- T10 spinal cord was injured with modified Allen’s method. | Contusion | Subacute | BMSCs | Tibia and femur of SD rats | Spinal cord | 14 days after operation | / | Normal saline |
| 101 | Fang 2017 | China | RCT | SD rats | Male | 200-220g | Adult | 20/20 | The compression plate (20g) was lowered down ventrally at a rate of 0.5 mm/min to the bottom of the vertebral canal and remained there for 5 min. | Extrusion | Acute | BMSCs | Tibia and femur of SD rats | Spinal cord | Immediately | 2×10^5^ | DMEM |
| 102 | Seo 2017 | Korea | RCT | SD rats | Female | 250-300g | Adult | 10/10 | A moderate spinal cord injury was induced by a 250kdyn force using an Infinite Horizon (IH) impactor (Precision System and Instrumentation, Fairfax, V A, USA). | Contusion | Subacute | UCMSCs | Human umbilical cord blood | Spinal cord | 7 days after operation | 1×10^6^ | PBS |
| 103 | OHTA 2017 | Japan | RCT | SD rats | Female | 210-230g | 10weeks | 8/5 | A 10-g weight was then dropped onto the spinal cord from a height of 25 mm. | Contusion | Subacute | ADMSCs | Back fat of SD rats | Caudal vein | 8 days after operation | 2.5×10^6^ | Normal saline |
| 104 | Min 2016 | Korea | Control | SD rats | Female | 250-280g | / | 8/5 | A spinal cord contusion injury was induced by a 200 kdyn force through the use of an infinite horizon (IH) impactor (Precision System and Instrumentation, Fairfax, VA, USA). | Contusion | Acute | ADMSCs | Inguinal adipose tissue of SD rats | Spinal cord | 3 days after operation | 1×10^7^ | PBS |
| 105 | Zhang 2017 | China | RCT | SD rats | Male | 250-350g | / | 5/5 | Transection of the spinal cord at the T12 level was performed using a taper scalpel to make a 2 mm transection injury split at spinal cord. | Transection | Subacute | BMSCs | Tibia and femur of SD rats | Spinal cord | 7 days after operation | 1×10^6^ | Blank |
| 106 | Nicola 2017 | Brazil | RCT | Wistar rats | Male | 200-250g | 8weeks | 8/8 | Laminectomy was performed at the level of 9th thoracic vertebra (T9) and injury was induced through the drop of a 10 g weight from 25 mm height by the use of New York University Impactor device. | Contusion | Acute | DPSCs | Deciduous tooth stem cells | Spinal cord | 1h after operation | / | Blank |
| 107 | Hwang 2014 | Korea | RCT | SD rats | Female | 250-300g | Adult | 6/6 | Animals were subjected to a contusion injury at the ninth thoracic vertebral level using the Infinite Horizon Impactor (200 kdyn). | Contusion | Subacute | NSCs | Spinal cord of embryonic SD rat | Spinal cord | 7 days after operation | 5×10^5^ | PBS |
| 108 | Zhou 2016 | China | RCT | Rats | / | / | / | 18/10 | Anesthesia by IP administration of 3.6% chloral hydrate (1 ml/100 g body weight), the skin was incised to expose the thoracic vertebral column, and a T9 laminectomy was performed. | Contusion | Acute | UCMSCs | Baby umbilical cord | Spinal cord | Immediately | 1×10^5^ | PBS |
| 109 | Asadi-Golshan 2018 | Iran | RCT | SD rats | Male | 250-280g | Adult | 10/10 | Laminectomy was performed at the thoracic level, and a compression injury was induced at T7 level by the application of a 20 g aneurysm clip (Harvard Apparatus) for 1 minute. | Extrusion | Acute | DPSCs | Human deciduous teeth | Spinal cord | Immediately | / | Blank |
| 110 | Sarveazad 2016 | Iran | RCT | Wistar rats | Male | 200-250g | Adult | 6/6 | Piston (weighing 10 g and 2 mm in diameter from a 12.5 cm distance) was released to cause moderate contusion SCI. | Contusion | Subacute | ADMSCs | Abdominal fat of adults | Spinal cord | 7 days after operation | 1×10^6^ | PBS |
| 111 | Melo 2016 | Brazil | RCT | Wistar rats | Male | / | 10weeks | 4–8 animals/group | An embolectomy catheter was subsequently introduced into the epidural space and inflated to 3.0 mm of diameter for 1 min. | Extrusion | Acute | hSDMSCs | The dermis of scalp tissue samples obtained from healthy female patients | Spinal cord | 1h after operation | 1×10^4^ | Blank |
| 112 | FENG 2021 | China | RCT | SD rats | Female | 220±20g | 8weeks | 12/12 | A 10‑g impactor was used to induce spinal cord contusion injury (100 g x cm x force) by the weight‑drop method. | Contusion | Subacute | BMSCs | Tibia and femur of SD rats | Spinal cord | 7 days after operation | 5×10^5^ | Blank |
| 113 | Ruzicka 2018 | Czech Republic | RCT | Wistar rats | / | 300±15g | 12weeks | 28/34 | The balloon was inflated with 15 μl of saline for 5 min to induce severe compression injury. | Extrusion | Acute | BMSCs | Human | Spinal cord | Immediately | 5×10^5^ | Normal saline |
| 114 | Bai 2019 | China | RCT | SD rats | Female | / | 6weeks | 10/10 | A weight of 10 g was dropped from a height of 5 cm onto the exposed spinal cord to cause moderate contusion at the T10 vertebrae. | Contusion | Acute | BMSCs | Bone marrow of SD rats | Spinal cord | Immediately | 1×10^6^ | Vehicle |
| 115 | Ohta 2018 | Japan | RCT | SD rats | Female | 210-230g | 10weeks | 8/10 | A 10 g weight was dropped onto the spinal cord from a height of 25 mm. | Contusion | Subacute | ADMSCs | Back fat of SD rats | Caudal vein | 8 days after operation | 2.5×10^6^ | Normal saline |
| 116 | Batista 2019 | Brazil | RCT | Wistar rats | Male | 250~300g | / | 12/14 | Animals were fixed by the upper and lower vertebrae and a moderate injury was inflicted by dropping a weight of 10 g from a 25-mm height. | Contusion | Subacute | NSCs | Wistar rat embryos | Spinal cord | 10 days after operation | 6×10^5^ | DMEM |
| 117 | Krupa 2018 | Czech Republic | RCT | Wistar rats | Male | 275-305g | Adult | 12/9/11 | The balloon center of the catheter rested at T8. With 15μL saline quickly inflated, keep 5min. | Extrusion | Subacute | UCMSCs | Baby umbilical cord | Spinal cord | 7 days after operation | 5×10^5^; 1.5×10^6^ | Normal saline |
| 118 | Kim 2018 | Korea | Control | SD rats | Male | 280-300g | / | 12/12 | A compression injury was made by applying a vascular clip (FD561R, BIEMER, Germany) for 15 min at the T9 level (30~40g of 0.29~0.39 N of closing force). | Extrusion | Acute | BMSCs | Human | Spinal cord | 3 days after operation | 1.5×10^5^ | PBS |
| 119 | LV 2019 | China | Control | SD rats | / | 180-200g | / | 5/5 | A 3.5 mm round plastic pad was placed on T10 spinal cord, and a 10 g iron cone was used to hit against the pad in a free fall at a height of 3 cm to cause impact injury to the spinal cord. | Contusion | Acute | BMSCs | Bone marrow of tibia in SD rats | Subarachnoid space | 30min after operation | 5×10^5^ | Blank |
| 120 | You 2019 | China | Control | SD rats | Male | 220±20g | Adult | 24/24 | A length of 2.0 mm of the T9 spinal cord level was removed. | Transection | Acute | BMSCs | Bone marrow of femur and tibia of SD rats | Spinal cord | Immediately | 1×10^6^ | Culture medium |
| 121 | Mohammadshirazi 2019 | Iran | Control | Wistar rats | Male | 250-280g | Adult | 9/12 | T9 and T11 spinous processes were fixed with clipless. The 10 g rod was released from 25 mm height on the exposed spinal cord via NYU‐impactor. | Contusion | Subacute | NSCs | / | / | 7 days after operation | 1×10^6^ | Blank |
| 122 | Sun 2019 | China | RCT | Wistar rats | Female | 180-210g | Adult | 25/25/25 | SCI model was created by dropping a 10-g weight rod from a height of 2.5 cm onto the exposed dorsal surface of the dura of the spinal cord with an NYU impactor. | Contusion | Subacute | NSCs; UCMSCs | Neonatal umbilical cord; Brain tissue of aborted fetus | Spinal cord | 7 days after operation | 6×10^5^ | PBS |
| 123 | Ma 2019 | China | RCT | SD rats | / | / | / | 9/9 | After a longitudinal incision preformed in the dura to expose the posterior median sulcus, the right half of the spinal cord was cut at T9 followed by the removal of a 2 mm segment. | Transection | Subacute | NSCs | Brain tissue of pregnant mouse embryos | Spinal cord | 7 days after operation | 5×10^5^ | Normal saline |
| 124 | Pang 2019 | China | RCT | Long-Evans hooded rats | Male | / | 11weeks | 6/6 | A 10-g rod equipped in an NYU/MASCIC impactor (NYU, USA) was dropped from a 25-mm height onto the T9–T10 spinal cord. | Contusion | Subacute | NSCs | Human wisdom teeth | Spinal cord | 7 days after operation | 1×10^6^ | Blank |
| 125 | Yuan 2019 | China | RCT | SD rats | Female | 200-250g | Adult | 30/30/25 | The dura was cut with an 11-blade scalpel, and then, a 2 mm long section of the spinal cord was completely removed at the T10 level using iridectomy scissors and microscope forceps under the dura. | Transection | Subacute | NSCs | Brain tissue of SD rats | Spinal cord | 7 days after operation | 2.5×10^4^; 1.5×10^5^ | PBS |
| 126 | Liu 2020 | China | RCT | SD rats | Female | 250±15g | 10weeks | 51/51/39 | The exposed dorsal surface of the T13 spinal segment was injured using a LISA impactor (Louisville Injury System Apparatus, Louisville, KY, USA) with a displacement of 1.0 mm for 0.5 seconds. | Contusion | Subacute | UCMSCs; ADMSCs | / | Spinal cord | 9 days after operation | 2.5×10^5^ | Culture medium |
| 127 | Yao 2020 | China | Control | SD rats | Female | 200-250g | Adult | 20/20 | the cell fibers were cut into 4 mm length and stacked to be implanted into the SCI lesion site. | Transection | Acute | BMSCs | / | Spinal cord | Immediately | 1×10^6^ | Blank |
| 128 | Du 2018 | China | RCT | SD rats | Male | 200-250g | Adult | 10/10 | SCI model was made by an ALLEN’S II impactor (MASCIS Impactor, W.M. Keck Center of Rutgers University, Piscataway, NJ, USA) with a dropping weight 10.0 g from 25-cm above the exposed cord. | Contusion | Acute | NSCs | Hippocampus of newborn rats | Spinal cord | Immediately | 2×10^5^ | Blank |
| 129 | Chudickova 2019 | Czech Republic | RCT | Wistar rats | Male | 250-300g | / | 18/20 | The balloon center of the catheter rested at T8. With 15μL saline quickly inflated, keep 5min. | Extrusion | Subacute | BMSCs | Neonatal umbilical cord | Spinal cord | 7 days after operation | 4×10^5^ | Normal saline |
| 130 | Salarinia 2020 | Iran | RCT | Wistar rats | Male | 220-240g | 12-14weeks | 12/12 | A 10 g metal rod was dropped onto the spinal cord from a 50 mm height. Finally, the incision was sutured. | Contusion | Subacute | ADMSCs | Fat tissue in inguinal region of Wistar rats | Spinal cord | 7 days after operation | 2×10^5^ | PBS |
| 131 | Wu 2020a | China | RCT | SD rats | Female | 230-250g | / | 30/30 | / | Not reported | Acute | UCMSCs | / | Spinal cord | 30min after operation | 1×10^5^ | DMSO |
| 132 | Moinuddin 2020 | America | RCT | SD rats | Female | 270-300g | / | 6/6 | / | Contusion | Subacute | UCMSCs | The umbilical cord of SD rats | Caudal vein | 7 days after operation | 2×10^6^ | Normal saline |
| 133 | Wang 2021 | China | RCT | SD rats | Female | 180-200g | 8weeks | 18/12 | The spinal cord was injured using a modified Allen’s impactor: a guided 9-g rod dropped 10 cm into the exposed dura matter to cause moderate SCI. | Contusion | Subacute | UCMSCs | Neonatal umbilical cord | Spinal cord | 7 days after operation | 5×10^6^ | Culture medium |
| 134 | Liao 2021 | China | RCT | SD rats | Female | 230±10g | / | 18/18 | The impactor (10-g rod, the diameter is 2 mm) was used to drop the small stick from a height of 7 cm onto the dorsal surface of the exposed T10 spinal cord after laminectomy. | Contusion | Acute | UCMSCs | / | Caudal vein | 1 days after operation | 1×10^5^ | PBS |
| 135 | Zarei-Kheirabadi 2020 | Iran | RCT | Wistar rats | Female | 250-280g | Adult | 10/10 | Laminectomy was performed by removing the thoracic vertebra at T10-11 level then contusion injury was generated using NYU Impactor (10 g, 25 mm). | Contusion | Subacute | ESCs | / | Spinal cord | 7 days after operation | 1×10^6^ | PBS |
| 136 | Zhou 2020 | China | RCT | SD rats | Female | 230-260g | Adult | 3/3 | The exposed dura mater at the T9 level was confused by a 10 g weight dropped from a height of 25 mm using an New York university impactor. | Contusion | Acute | hAMSCs | Placenta of healthy women | Caudal vein | 2h after operation | 4×10^6^ | PBS |
| 137 | Deng 2021 | China | RCT | SD rats | Female | 200-250g | 8weeks | 8/8 | The SCI strike device was used to hit the spines (impact rod weight: 10 g; drop height: 2.5 cm; contact time: 0.1 s) caused SCI to the rats. | Contusion | Acute | NSCs | Rat embryonic hippocampal tissue | Spinal cord | Immediately | 1×10^6^ | Normal saline |
| 138 | Hamidabadi 2021 | Iran | RCT | SD rats | Male | 200-250g | / | 8/8 | A metal rod of 10 g (with a diameter of 2 mm) was dropped from a height of 25 mm to induce contusive SCI. | Contusion | Acute | hOE-MSCs | Patients’ nasal mucosa | Spinal cord | 1 days after operation | 5×10^5^ | PBS |
| 139 | Wu 2020b | China | Control | ICRmice | / | 35-47g | 15weeks | 8/8 | The M-III Spinal Impactor (W.M. Keck Center for Collaborative Neuroscience) was used to induce contusive SCI by applying 90 kilodynes of force to the exposed spinal cord. | Contusion | Subacute | UCMSCs | / | Spinal cord | 7 days after operation | 1×10^5^ | Culture medium |
| 140 | Li 2021 | China | RCT | SD rats | Male | 220-250g | 8weeks | 17/17 | The spinal cord was compressed using a calibrated aneurysm clip, which provided 20 g/cm2 pressure. The clip was released after 60 sec. | Extrusion | Acute | NSCs | Brain tissue of SD rat embryos | Spinal cord | Immediately | 2×10^5^ | DMEM |
| 141 | Kang 2015 | China | RCT | SD rats | Half male and half female | 200-250g | / | 12/12 | Cut through the right spinal cord using an iridectome and cut out 1 mm long spinal cord from the ends. | Transection | Acute | NSCs | Embryos of SD rats | Spinal cord | Immediately | 1×10^6^ | Normal saline |
| 142 | Xiong 2017 | China | RCT | SD rats | Male | 220±20g | Adult | 15/15 | Complete transection of the T10 spinal cord was performed and the intervening tissue was removed. | Transection | Subacute | HSCs | Bone marrow of SD rats | Spinal cord | 7 days after operation | 1.2×10^5^ | DMEM |
| 143 | Kumar 2018 | India | RCT | Wistar rats | Male | / | Adult | 15/17 | The spinal cord was exposed after laminectomy and then transected (T11 vertebra) completely by a fine dura microscissors. | Transection | Subacute | BMSCs | Bone marrow of SD rats | Spinal cord | 9 days after operation | 2.5×10^5^ | PBS |
| 144 | Zhang 2018 | China | RCT | SD rats | / | 180-220g | Adult | 6/6 | A force of 200 kilodynes was induced an impact injury to the spinal cord using the PSI–IH Impactor, which has sensors to accurately measure the impact force. | Contusion | Chronicity | BMSCs | Bone marrow of SD rats | Spinal cord | 4 weeks after operation | 4×10^5^;8×10^5^;1×10^6^ | DMEM |
| 145 | Chen 2012 | China | RCT | SD rats | Male | 150-250g | 8-12weeks | 10/10 | According to the Allen method with some modifications, the T10 segment of the spinal cord was exposed for blast injury under the force of 50 g·cm. | Contusion | Acute | BMSCs | Bone marrow of SD rats | Caudal vein | 3 days after operation | 1×10^6^ | PBS |
| 146 | Wang 2016 | China | RCT | SD rats | Female | 250-300g | Adult | 10/6 | A hemi section was performed in the spinal cord at the left T11 level, and the partial spinal tissue at the hemi section site was aspirated to create a cavity 3 mm in diameter. | Transection | Subacute | AECs | Placenta of pregnant women | Spinal cord | 7 days after operation | 1×10^4^ | Culture medium |
| 147 | Lei Li 2007 | China | RCT | Wistar rats | Half male and half female | 250-350g | 16weeks | 30/30 | The no. 11 blade creates a semi-transection of the spinal cord at the left. | Transection | Acute | BMSCs | Tibia and femur of SD rats | Spinal cord | Immediately | 6×10^5^ | PBS |
| 148 | Wenli Jing 2008 | China | RCT | SD rats | Male | 260-280g | Adult | 12/12 | T12 spinal cord contusion injury was caused when a 10g stainless steel rod was dropped 7.5cm from the three-dimensional Wendi by self-made improved Allen device. | Contusion | Subacute | BMSCs | Tibia and femur of SD rats | Spinal cord; Caudal vein | 7 days after operation | 1×10^6^ | Blank |
| 149 | Yutao Jia 2020 | China | RCT | SD rats | Male | 200±10g | 8weeks | 20/20 | A modified Allen's strike (10g) is used to hit the exposed area at a height of 2.5cm. | Contusion | Acute | BMSCs | Tibia and femur of SD rats | Caudal vein | 1 days after operation | 2×10^6^ | Normal saline |
| 150 | Jun Zhou 2011 | China | RCT | SD rats | Male | 250-319g | Adult | 16/16 | The animal model of spinal cord injury was established by modified Allen method. | Contusion | Subacute | BMSCs | Tibia and femur of SD rats | Subarachnoid space | 7 days after operation | 1×10^6^ | PBS |
| 151 | Jingsong Sun 2018 | China | RCT | SD rats | Male | 180±5g | 12weeks | 10/10 | After the T10 spinal cord was exposed, a large vein was seen in the dorsal middle of the spinal cord. The spinal cord was clamped to 1/2 of its original diameter with an aneurysm clamp for about 30s. | Ischemia | Acute | BMSCs | Tibia and femur of SD rats | Caudal vein | Immediately | 2×10^6^ | Blank |
| 152 | Jiajia Sun 2016 | China | RCT | SD rats | Male | 260-300g | 6weeks | 16/16 | Drop smooth metal bar of 2.4mm, diameter 10g and drop from 5cm. | Contusion | Subacute | BMSCs | Tibia and femur of SD rats | Subarachnoid space | 7 days after operation | 1×10^6^ | PBS |
| 153 | Cuiping Mo 2015 | China | RCT | SD rats | Female | 250-300g | 10weeks | 5/5 | Causing a 40g impacting rod to fall free from a height of 5cm with an injury energy of 200g/cm, impacting the spinal cord corresponding to the T10 bone window, resulting in acute spinal cord injury. | Contusion | Acute | BMSCs | Tibia and femur of SD rats | Spinal cord | Immediately | 1×10^6^ | PBS |
| 154 | Xuezheng Zhao 2015 | China | RCT | SD rats | Female | 220-250g | / | 12/12 | Using a modified Allen's strike, a 2g stainless steel rod dropped 12.5cm (25g·cm) freely from the three-dimensional stereoscope, resulting in contusion and laceration of T10 spinal cord. | Contusion | Acute | BMSCs | Tibia and femur of SD rats | Spinal cord | 3 days after operation | 1.5×10^5^ | PBS |
| 155 | Kaijun Liu 2008 | China | RCT | SD rats | Half male and half female | 250-280g | / | 15/15 | A rat model of spinal cord complete transection injury was established by modified Tuszynsl method  at T9 level. | Transection | Acute | BMSCs | Tibia and femur of SD rats | Caudal vein | Immediately | 1×10^6^ | PBS |
| 156 | Hongtao Zhang 2007 | China | RCT | Wistar rats | Half male and half female | 195-285g | Adult | 20/20 | The pier of a 55 g weight weight (0.4cm × 0.3cm) was placed on the surface of the dura mater for 1 min, and the spinal cord was injured by compression. | Extrusion | Subacute | UCMSCs | Neonatal umbilical cord | Saphenous vein | 5 days after operation | 1×10^6^ | Normal saline |
| 157 | Daxiong Feng 2009 | China | RCT | SD rats | Female | 100-150g | 6weeks | 22/22 | Through self-made Allen striking rack, to rat TAs the center, the striking potential energy is 50gcm, and the Allen model of total spinal cord paralysis caused by heavy percussion is made. | Contusion | Subacute | BMSCs | SD rats | Caudal vein | 7 days after operation | 2×10^6^ | Culture medium |
| 158 | Jianhua Lin 2005 | China | RCT | SD rats | Half male and half female | 300g | / | 14/10 | A circular thin copper gasket (with 3mm diameter of 7.075 square mm, weight of 0.1g) was placed on the surface of the T10 segment spinal cord Strike the gasket freely from 5 cm at a weight of 10g. | Contusion | Acute | BMSCs | Tibia and femur of SD rats | Caudal vein | 3 days after operation | 1×10^6^ | PBS |
| 159 | Yuliang Liu 2015 | China | RCT | Wistar rats | Half male and half female | 200-220g | / | 20/20/10 | The model of spinal cord injury was developed by modified Allen method. | Contusion | Acute | UCMSCs | Neonatal umbilical cord | Spinal cord; Caudal vein | Immediately | 2×10^5^ | Blank |
| 160 | Cheng Liu 2018 | China | RCT | SD rats | / | 200-300g | / | 20/20 | A rat SCI model was made using a modified heavy object strike method. | Contusion | Subacute | UCMSCs | / | Spinal cord; Caudal vein | 7 days after operation | 2×10^6^ | PBS |
| 161 | Yuekui Wu 2014 | China | RCT | SD rats | Female | 180-200g | Adult | 20/20 | / | Not reported | Subacute | NSCs | Cord blood of pregnant women | Spinal cord | 7 days after operation | 1×10^7^ | PBS |
| 162 | Ming Deng 2018 | China | RCT | SD rats | Half male and half female | 250-270g | 6-8weeks | 12/12 | Allen method was used to fix a 10 g hammer at a height of 7 cm above the spinal cord (injury was 70 g/cm). | Contusion | Acute | hUSCs | Urine of adults | Spinal cord | 1 days after operation | 2×10^5^ | DMEM |
| 163 | Huihui Dou 2010 | China | RCT | SD rats | Half male and half female | 200g | / | 10/10 | / | Not reported | Acute | UCMSCs | Neonatal umbilical cord | Caudal vein | 3 days after operation | 5×10^6^ | DMEM |
| 164 | Huangfei Yu 2012 | China | RCT | SD rats | Female | 220±20g | Adult | 20/20 | Horizontal cord was performed at the T10 level. | Transection | Acute | hAMSCs | Placenta of pregnant women | Spinal cord | Immediately | 2×10^5^ | PBS |
| 165 | Zhe Li 2014 | China | RCT | SD rats | Male | 250±25g | Adult | 30/30 | SCI rat model was made by modified Allen’s method. | Contusion | Acute | NSCs | Brain tissue of neonatal rats | Caudal vein | 1 days after operation | 5×10^6^ | PBS |
| 166 | Zhe Li 2013 | China | RCT | SD rats | Half male and half female | 200±50g | Adult | 30/30 | Spinal cord injury by modified Allen’s striking method (25 cm·g= 10 g×2.5 cm, i.e. the force of injury caused by 10 g weight falling vertically from 2.5 cm height). | Contusion | Chronicity | NSCs | Rat embryonic hippocampal tissue | Caudal vein; Spinal cord | 4 weeks after operation | 5×10^6^ | Blank |
| 167 | Ning LIu 2011 | China | RCT | SD rats | Male | 220-250g | / | 12/12 | A 2mm wide spinal cord was excised by elbow microshear to confirm complete spinal cord transection. | Transection | Subacute | NSCs | Brain tissue of neonatal rats | Spinal cord | 7 days after operation | 1×10^4^ | DMEM |
| 168 | Jieliang Hu 2014 | China | RCT | SD rats | Half male and half female | 200-250g | / | 12/12 | The right spinal cord was cut by iris knife and 1mm spinal cord was cut at the severed end. | Transection | Acute | NSCs | / | Spinal cord | Immediately | 1×10^6^ | Normal saline |
| 169 | Chaohui Cheng 2007 | China | RCT | SD rats | / | 300±15g | 7weeks | 12/12 | By impinging the dorsal side of the spinal cord with a self-made spinal cord injury striking device (iron rod weighing 10g, dropping 2.5cm, diameter of the lower end of the rod 0.25mm). | Contusion | Acute; Subacute | BMSCs | Bone marrow of human iliac bone | Spinal cord | Immediately;7 days after operation | 2.5×10^5^ | Blank |
| 170 | Mingyuan Han 2010 | China | RCT | Wistar rats | Female | 230±10g | 8weeks | 20/10 | The model of spinal cord injury in rats was established by modified Allen’s method. | Contusion | Subacute | UCMSCs | Neonatal umbilical cord | Subarachnoid space | 7 days after operation | 1×10^6^ | Blank |
| 171 | Zhiying Li 2009 | China | RCT | SD rats | Half male and half female | 240±10g | 10weeks | 30/30 | According to NYU spinal cord injury strike method, 25mm height, 10g mass, free fall strike was selected. | Contusion | Acute | BMSCs | Bone marrow of SD rats | Spinal cord | 1h after operation | 3×10^6^ | Normal saline |
| 172 | Zhongjie Shen 2020 | China | RCT | SD rats | Male | / | / | 18/18 | SCI model was prepared by using the modified Allen method (40g weight struck vertically from 5cm height). | Contusion | Acute | BMSCs | Bone marrow of the femur of the SD rat | Spinal cord | Immediately | 2.5×10^4^ | PBS |
| 173 | Jianping Cheng 2019 | China | RCT | SD rats | Female | 250g | 6weeks | 13/14 | Allen's percussion method to establish spinal cord injury model. | Contusion | Acute | BMSCs | Femur and tibia of newborn rats | Spinal cord | Immediately | 1.5×10^5^ | Blank |
| 174 | Zhijun Zhao 2013 | China | RCT | SD rats | / | / | / | 20/20 | Allen's percussion method to establish spinal cord injury model. | Contusion | Acute | NSCs | Brain tissue of neonatal SD rats | Spinal cord | 6h after operation | 1×10^5^ | Blank |
| 175 | Jianwei Sun 2020 | China | RCT | SD rats | Female | 280±20g | 8weeks | 15/15 | Allen method was used for modeling, and a self-made 10g weight hit the spacer covering the spinal cord surface with a free fall of 5cm height. | Contusion | Acute | BMSCs | Femur and tibia of SD rats | Spinal cord | 1h after operation | 1.5×10^5^ | Normal saline |
| 176 | Deshui Yu 2011 | China | RCT | Wistar rats | Female | 220-250g | Adult | 33/33 | The spinal cord was vertically compressed for 5min with a weight of 35g and a contact area of 4mm2, taking the T9 relative area as the injury area and the posterior median blood vessel as the center. | Contusion | Acute | BMSCs | Femur and tibia of SD rats | Spinal cord | 0.5h after operation | 3×10^5^ | DMEM |
| 177 | Zhiyong Pu 2018 | China | RCT | SD rats | Half male and half female | 250-350g | 3-4weeks | 36/36 | The spinal cord was struck with a force of 10 g×5 cm using the Allen percussion device. | Contusion | Acute | BMSCs | Femur and tibia of SD rats | Spinal cord | Immediately | 6×10^6^ | PBS |
| 178 | Yu Feng 2013 | China | RCT | SD rats | Female | 220-250g | 7-9weeks | 15/15 | Compression injury of spinal cord was caused by contact with a homemade plexiglass rod with a mass of 100g and a diameter of 2.0mm, which was removed after 3min. | Contusion | Acute | BMSCs | Femur and tibia of SD rats | Spinal cord | 0.5h after operation | 1.5×10^5^ | DMEM |
| 179 | Houyong Yan 2017 | China | RCT | SD rats | Female | 250-300g | Adult | 20/20 | The 5G striking rod was free falling from a height of 5cm through the sleeve to hit the plastic spacer. | Contusion | Acute | NSCs | / | Subarachnoid space | 6h after operation | 1×10^5^ | Normal saline |
| 180 | Zonghao Zhu 2014 | China | RCT | SD rats | Male | 220-250g | Adult | 15/15 | The right half of the spinal cord was transected by transverse puncture at the right half of the T8 spinal cord with microscissors. | Transection | Subacute | NSCs | Embryos of SD rats | Spinal cord | 7 days after operation | 1×10^6^ | Blank |
| 181 | Dabin Wang 2010 | China | RCT | SD rats | Female | 250±50g | Adult | 10/10 | The dural was dissected in the middle of the spinal cord, and the spinal cord 4mm to the right of T9 level was resected. | Transection | Acute | BMSCs | Femur and tibia of SD rats | Spinal cord | Immediately | 2×10^5^ | Blank |
| 182 | Amemori 2013 | Czech Republic | RCT | Wistar rats | Male | 270-300g | 10weeks | 20/16 | The balloon center of the catheter rested at T8. With 15μL saline quickly inflated, keep 5min. | Extrusion | Subacute | NSCs | Human fetal spinal cord | Spinal cord | 7 days after operation | 5×10^5^ | Blank |
| 183 | Amemori 2015 | Czech Republic | RCT | Wistar rats | Male | 270-300g | Adult | 9/9 | A sterile 2french Fogarty catheter was inserted into the epidural space until the center of the balloon rested on thoracic vertebra 8 (T8). The balloon was rapidly inflated with 15μl saline for 5 minutes. | Extrusion | Subacute | NSCs | Human fetal lung fibroblasts | Spinal cord | 7 days after operation | 5×10^5^ | Normal saline |
| 184 | Du 2011 | China | RCT | SD rats | Female | 220-250g | Adult | 5/5 | Following laminectomy at the T9 vertebral level, the spinal cord was transected and a 2-mm cord segment including visible spinal roots was completely removed at the T10 spinal cord level. | Transection | Acute | NSCs | Hippocampus of SD Rats | Spinal cord | Immediately | / | Blank |
| 185 | Edalat 2013 | Iran | RCT | SD rats | Female | 200-250g | Adult | 10/10 | A 10-g impact rod (with a 2 mm diameter) was dropped from a height of 25 mm in order to produce contusive SCI. | Contusion | Subacute | BMSCs | Femur and tibia of SD rats | Spinal cord | 7 days after operation | 5×10^5^ | PBS |
| 186 | Maeda 2009 | Japan | RCT | Wistar rats | Male | 250-300g | Adult | 8/8 | The exposed spinal cords were contused by driving the rack downward at 1.20–1.30 m/s using pneumatic injury device (PiD-1000, Physio- Tech, Tokyo, Japan). | Contusion | Subacute | NSCs | The hippocampus of Fischer rats | Spinal cord | 7 days after operation | 1×10^5^ | Culture medium |
| 187 | Rosado 2017 | Brazil | RCT | Wistar rats | / | 300g | / | 50/20 | After visualization of the spinal cord covered by intact dura mater, compressive SCI was induced using a French Fogarty No. 2 catheter at T10. | Contusion | Acute | ADMSCs | GFP Lewis rat adipose tissue | Caudal vein | 3h after operation | 1×10^6^ | PBS |
| 188 | Tang 2015 | China | RCT | SD rats | Female | 220-250g | Adult | 16/16 | A metal rod with a weight of 25 g and a diameter of 2 mm was dropped from a height of 3 cm onto the exposed spinal cord to induce a contusion lesion at T8-T9. | Contusion | Subacute | ADMSCs | Adipose tissue of SD rats | Spinal cord | 9 days after operation | 1×10^6^ | DMEM |

**Table 3: Traditional Meta - analysis results of 1 week after stem cell therapy**

| **Interventions** | **WMD** | **Number** | **I-squared** | **Z** | **P** |
| --- | --- | --- | --- | --- | --- |
| UCMSCs | 1.248 [0.799, 1.696] | 21 | 94.30% | 5.45 | 0 |
| ADMSCs | 1.133 [0.339, 1.927] | 10 | 88.60% | 2.8 | 0.005 |
| NSCs | 0.862 [0.642, 1.083] | 31 | 94.90% | 7.68 | 0 |
| BMSCs | 1.258 [0.998, 1.517] | 84 | 97.30% | 9.49 | 0 |
| HSCs | 1.300 [0.521, 2.079] | 1 | / | 3.27 | 0.001 |
| DPSCs | 3.098 [0.943, 5.252] | 5 | 97.80% | 2.82 | 0.005 |
| PDMSCs | 5.050 [4.366, 5.734] | 1 | / | 14.47 | 0 |
| AECs | 1.700 [0.988, 2.412] | 2 | 0.00% | 4.68 | 0 |
| ASCs | 3.570 [2.750, 4.390] | 1 | / | 8.53 | 0 |
| IPSCs | 3.330 [3.067, 3.593] | 1 | / | 24.77 | 0 |
| hSDMSCs | 1.520 [0.597, 2.443] | 1 | / | 3.23 | 0.001 |
| ESCs | 1.460 [0.864, 2.056] | 1 | / | 4.8 | 0 |
| hAMSCs | 0.437 [0.128, 0.747] | 2 | 0.00% | 2.77 | 0.006 |

**Table 4:** **Traditional Meta-analysis results of 3 week after stem cell therapy**

| **Interventions** | **WMD** | **Number** | **I-squared** | **Z** | **P** |
| --- | --- | --- | --- | --- | --- |
| ADMSCs | 3.33 [2.082, 4.584] | 11 | 95.60% | 5.22 | 0 |
| BMSCs | 2.790 [2.404, 3.176] | 75 | 96.50% | 14.16 | 0 |
| NSCs | 2.437 [1.925, 2.948] | 27 | 95.90% | 9.33 | 0 |
| UCMSCs | 3.048 [2.353, 3.743] | 21 | 97.60% | 8.59 | 0 |
| HSCs | 1.885 [1.438, 2.332] | 2 | 0.00% | 8.26 | 0 |
| DPSCs | 3.492 [2.381, 4.604] | 5 | 89.30% | 6.16 | 0 |
| PDMSCs | 7.890 [7.411, 8.369] | 1 | / | 32.31 | 0 |
| AECs | 4.600 [3.695, 5.505] | 2 | 0.00% | 9.96 | 0 |
| ASCs | 1.430 [0.982, 1.878] | 1 | / | 6.26 | 0 |
| IPSCs | 4.490 [4.252, 4.728] | 1 | / | 36.95 | 0 |
| hSDMSCs | 3.620 [2.643, 4.597] | 1 | / | 7.26 | 0 |
| ESCs | 2.590 [1.851, 3.329] | 1 | / | 6.87 | 0 |
| hAMSCs | 3.853 [3.120, 4.586] | 2 | 71.40% | 10.3 | 0 |
| hOE-MSCs | 3.120 [2.383, 3.857] | 1 | / | 8.3 | 0 |
| hUSCs | 4.760 [3.437, 6.083] | 1 | / | 7.05 | 0 |

**Table 5:** **Traditional Meta-analysis results of 5 week after stem cell therapy**

| **Interventions** | **WMD** | **Number** | **I-squared** | **Z** | **P** |
| --- | --- | --- | --- | --- | --- |
| ADMSCs | 3.722[2.323,5.121] | 8 | 95.30% | 5.22 | 0 |
| BMSCs | 3.483[2.953,4.012] | 44 | 97.80% | 12.9 | 0 |
| NSCs | 2.664[2.228,3.100] | 23 | 94.50% | 11.98 | 0 |
| UCMSCs | 3.599[2.448,4.751] | 14 | 99.00% | 6.13 | 0 |
| HSCs | 2.610[1.863,3.357] | 1 | / | 6.85 | 0 |
| DPSCs | 4.709[2.880,6.539] | 5 | 95.80% | 5.04 | 0 |
| ASCs | 1.800[1.246,2.354] | 1 | / | 6.36 | 0 |
| IPSCs | 5.020[4.811,5.229] | 1 | / | 47 | 0 |
| hSDMSCs | 3.940[2.901,4.979] | 1 | / | 7.43 | 0 |
| ESCs | 1.810[1.016,2.604] | 1 | / | 4.47 | 0 |
| hOE-MSCs | 5.020 [3.802, 6.238] | 1 | / | 8.08 | 0 |
| hAMSCs | 4.780[4.399,5.161] | 1 | / | 21.57 | 0 |

**Table 6:** **Traditional Meta-analysis results of 8 week after stem cell therapy**

| **Interventions** | **WMD** | **Number** | **I-squared** | **Z** | **P** |
| --- | --- | --- | --- | --- | --- |
| ADMSCs | 4.288 [1.033,7.543] | 3 | 97.00% | 2.58 | 0.01 |
| BMSCs | 3.197 [2.545,3.849] | 22 | 97.40% | 9.61 | 0 |
| NSCs | 3.414 [2.951,3.878] | 18 | 98.20% | 14.45 | 0 |
| UCMSCs | 3.330 [2.041,4.619] | 12 | 98.90% | 5.06 | 0 |
| DPSCs | 7.460 [6.915,8.005] | 1 | / | 26.83 | 0 |
| ASCs | 2.190 [1.514,2.866] | 1 | / | 6.35 | 0 |
| IPSCs | 5.510 [5.289,5.731] | 1 | / | 48.95 | 0 |
| hSDMSCs | 2.980 [1.574,4.386] | 1 | / | 4.15 | 0 |
| hOE-MSCs | 5.830 [4.584, 7.076] | 1 | / | 9.17 | 0 |
| hAMSCs | 4.580 [4.080,5.080] | 1 | / | 17.96 | 0 |

**Table 7:** **Ranking plot of therapeutic potential in the first week after stem cell therapy**

| Drug | Rank 1 | Rank 2 | Rank 3 | Rank 4 | Rank 5 |
| --- | --- | --- | --- | --- | --- |
| ADMSCs | 0.34 | 0.23 | 0.25 | 0.17 | 0.01 |
| BMSCs | 0.18 | 0.41 | 0.37 | 0.04 | 0 |
| NSCs | 0.02 | 0.05 | 0.21 | 0.71 | 0.01 |
| Placebo | 0 | 0 | 0 | 0.02 | 0.98 |
| UCMSCs | 0.47 | 0.3 | 0.17 | 0.05 | 0 |

**Table 8:** **Ranking plot of therapeutic potential in the third week after stem cell therapy**

| **Drug** | **Rank 1** | **Rank 2** | **Rank 3** | **Rank 4** | **Rank 5** |
| --- | --- | --- | --- | --- | --- |
| ADMSCs | 0.4 | 0.18 | 0.18 | 0.24 | 0 |
| BMSCs | 0.25 | 0.38 | 0.28 | 0.09 | 0 |
| NSCs | 0.1 | 0.17 | 0.27 | 0.46 | 0 |
| Placebo | 0 | 0 | 0 | 0 | 1 |
| UCMSCs | 0.26 | 0.27 | 0.26 | 0.21 | 0 |

**Table 9: Ranking plot of therapeutic potential in the fifth week after stem cell therapy**

| **Drug** | **Rank 1** | **Rank 2** | **Rank 3** | **Rank 4** | **Rank 5** |
| --- | --- | --- | --- | --- | --- |
| ADMSCs | 0.47 | 0.22 | 0.2 | 0.11 | 0 |
| BMSCs | 0.22 | 0.4 | 0.33 | 0.04 | 0 |
| NSCs | 0.01 | 0.05 | 0.18 | 0.76 | 0 |
| Placebo | 0 | 0 | 0 | 0 | 1 |
| UCMSCs | 0.29 | 0.33 | 0.29 | 0.1 | 0 |

**Table 10: Ranking plot of therapeutic potential in the eighth week after stem cell therapy**

| **Drug** | **Rank 1** | **Rank 2** | **Rank 3** | **Rank 4** | **Rank 5** |
| --- | --- | --- | --- | --- | --- |
| ADMSCs | 0.64 | 0.14 | 0.09 | 0.14 | 0 |
| BMSCs | 0.05 | 0.19 | 0.35 | 0.41 | 0 |
| NSCs | 0.1 | 0.29 | 0.34 | 0.28 | 0 |
| Placebo | 0 | 0 | 0 | 0 | 1 |
| UCMSCs | 0.22 | 0.38 | 0.22 | 0.18 | 0 |
